# Effect of Interlayer Bonding on Superlubric Sliding of Graphene Contacts: A Machine-Learning Potential Study

**DOI:** 10.1021/acsnano.3c13099

**Published:** 2024-03-28

**Authors:** Penghua Ying, Amir Natan, Oded Hod, Michael Urbakh

**Affiliations:** † Department of Physical Chemistry, School of Chemistry, The Raymond and Beverly Sackler Faculty of Exact Sciences and The Sackler Center for Computational Molecular and Materials Science, Tel Aviv University, Tel Aviv 6997801, Israel; ‡ Department of Physical Electronics, Tel Aviv University, Tel Aviv 6997801, Israel

**Keywords:** Machine-learning potentials, Graphene interfaces, Structural superlubricity, Interlayer bonding, Molecular dynamics, Atomic defects, Nanoscale friction

## Abstract

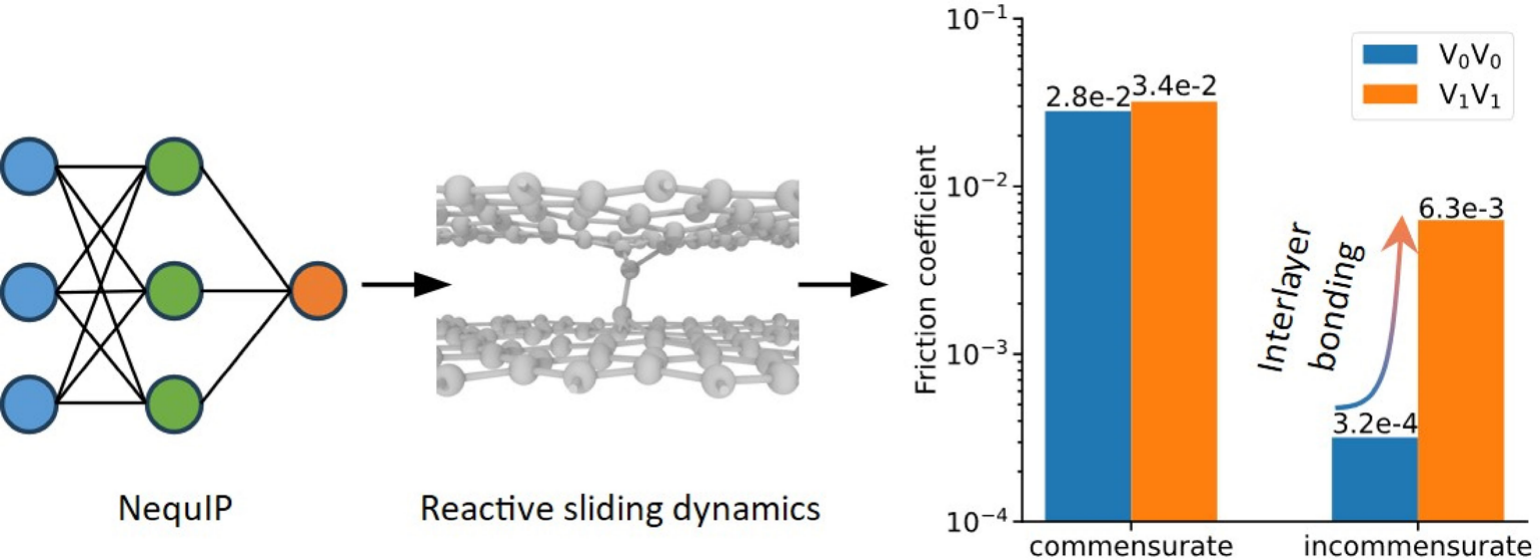

Surface defects and their mutual interactions are anticipated to affect the superlubric sliding of incommensurate layered material interfaces. Atomistic understanding of this phenomenon is limited due to the high computational cost of ab initio simulations and the absence of reliable classical force-fields for molecular dynamics simulations of defected systems. To address this, we present a machine-learning potential (MLP) for bilayer defected graphene, utilizing state-of-the-art graph neural networks trained against many-body dispersion corrected density functional theory calculations under iterative configuration space exploration. The developed MLP is utilized to study the impact of interlayer bonding on the friction of bilayer defected graphene interfaces. While a mild effect on the sliding dynamics of aligned graphene interfaces is observed, the friction coefficients of incommensurate graphene interfaces are found to significantly increase due to interlayer bonding, nearly pushing the system out of the superlubric regime. The methodology utilized herein is of general nature and can be adapted to describe other homogeneous and heterogeneous defected layered material interfaces.

In the last two decades, significant advancements in comprehending the atomic mechanisms underlying friction have led to the observation of ultralow, and even near-zero, friction (with friction coefficients below 10^–3^–10^–4^) at incommensurate microscale interfaces of two-dimensional (2D) materials, a phenomenon known as structural superlubricity.^1−5^ Scaling up this phenomenon toward the meso- and macroscales bears great technological potential for the reduction of energy loss and material wear in (electro-)mechanical devices. This, however, inevitably implies the appearance of multigrain surfaces with corrugated grain boundaries and intrinsic surface defects, which may introduce additional energy dissipation routes and eliminate superlubricity. Recently, the mechanisms of friction at grain boundaries have been studied theoretically, computationally, and experimentally, demonstrating counterintuitive phenomena including negative differential friction coefficient behavior, where friction reduces with external load.^6^ Lattice defects, such as vacancies, Stone–Wales defects, and surface edges, however, may hinder superlubricity via other mechanisms, such as elastic pinning and interlayer covalent bond formation.^1,7^ Understanding the microscopic origin of such phenomena, while being highly desirable, is challenging in practice due to the complex reactive dynamics invoked by such defects.^8^

In principle, ab initio simulations provide the desired toolset for meeting this challenge.^9,10^ Nonetheless, due to their computational demand, such studies are limited to relatively small model systems and short time-scales. Molecular dynamics (MD) simulations based on classical force-fields, parametrized against first-principles calculations for specific model systems, present an alternative that often balances well between computational efficiency and physical accuracy for the description of the anisotropic nature of the interactions in pristine layered interfaces.^11−21^ Such force-fields, however, must be hand-tailored to describe desired interactions and phenomena, where the inclusion of ingredients required to describe dynamical bond formation and rupture is highly nontrivial, especially under shear motion and the burden of normal load. To meet this need, one may resort to the emerging technology of machine-learning potentials (MLPs),^22−25^ which circumvents the need for explicit physical expressions to describe specific interactions and translates ab initio data directly into classical interatomic forces. This does not come without a price, as the physical understanding of the interatomic interactions is lost in the process while allowing the treatment of highly complex chemically reactive scenarios. Recent progress in the field of MLPs has enabled the modeling of reactive material processes, such as the proton transport in titanium dioxide-water interfaces,^26^ deposition of tetrahedral amorphous carbon,^27,28^ and the description of the phase diagrams of water^29^ and dense hydrogen.^30^

MLPs primarily comprise a complex functional to describe high-dimensional potential energy surfaces (PESs), which is trained against ab initio data (typically density functional theory (DFT) total energies and their spatial gradients) of a set of structural configurations. Often, the atomic positions are first translated into a set of descriptors (e.g., interatomic distances, angles, and other symmetry functions), which obey desired behavior under specific symmetry operations, such as invariance of energy under rotation, translation, and permutation of identical atoms.^34^ Different learning approaches include linear regression,^31,32^ kernel-based methods,^34,33^ and neural networks (NNs).^35,36^ Recently, graph neural network (GNN) interatomic potentials^22^ became popular due to their superior accuracy over previous MLP models in describing the interactions in small molecules, amorphous carbon, and liquid water. This can be attributed to their message-passing architectures and equivariant feature representations for the atomic environments.^37−39^

In the present study, we develop a GNN potential for bilayer defected graphene based on the neural equivariant interatomic potential (NequIP) scheme.^38^ Using an iterative learning approach, we explore the vast space of sliding configurations, including interlayer bonding scenarios. We consider the sliding dynamics of both aligned (commensurate) and twisted (incommensurate) interfaces, showing a relatively mild defect-induced increase of the coefficient of friction (COF) for the former and a significant COF increase for the latter to the extent of pushing the system nearly out of the superlubric regime.

## Results and Discussion

**Figure 1:**
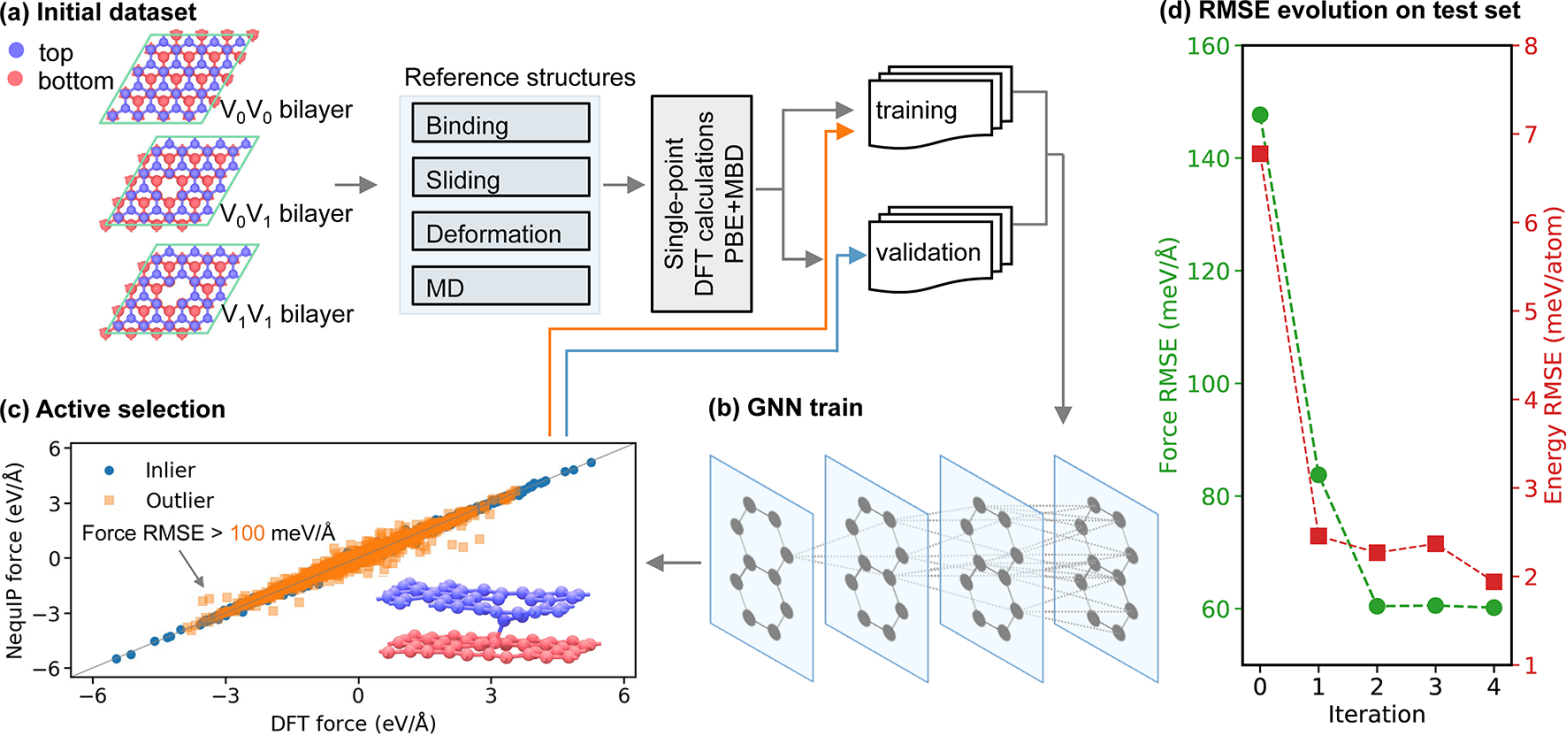
Schematic representation of the iterative process applied to construct the NequIP model. (a) The initial reference structures were obtained from MD simulations and manual manipulations for binding, sliding, and deformation scenarios. Single point DFT (PBE+MBD) calculations were performed for selected structures, forming training and validation sets. (b) Schematic diagram of the message-passing algorithm in the GNN potential. (c) Demonstration of active selection of outlier structures (a representative structure is shown in the inset) in V_1_V_1_ (having a single vacancy in each layer) bilayer sliding dynamics to be included in the next training iteration. (d) Illustration of the evolution of the test set force and energy RMSEs with iteration cycle.

Our iterative learning NequIP approach for defected bilayer graphene is illustrated in Figure 1. Three bilayer systems denoted by V_0_V_0_ (pristine bilayers), V_0_V_1_ (a single vacancy on the top layer and a pristine bottom layer), and V_1_V_1_ (a single vacancy in each layer) were used as reference structures (see Figure 1a). The initial reference data set included single-point total DFT (PBE+MBD, see Methods section) energies and atomic forces obtained for different interlayer distances, aligned stacking modes, manually deformed structures, and snapshot configurations taken from classical (for V_0_V_0_) and DFT-based (for the defected contacts) MD simulations under different temperatures (see Supporting Information (SI) section S1 for further details). This reference data set was split into two groups, namely, training and validation. The former subset served to train the MLP, whereas the latter subset served to monitor the error for preventing overfitting. The splitting was based on farthest point sampling (FPS),^36,40,41^ by performing a principal components analysis and choosing a set of sufficiently distant random points to serve as the training set and the remaining points to serve as the validation set (see SI section S2 for further details).

After obtaining the initial NequIP model, we initiated the iterative learning process, where we performed reactive sliding dynamics MD simulations of the V_1_V_1_ bilayer system, under different normal loads, using the present NequIP model (see Methods and SI section S3). In each iteration, 200 snapshots were extracted from the MD trajectory, and their total energy and force root-mean-square errors (RMSEs) relative to DFT results were calculated. Structures with force RMSE greater than 100 meV/Å were added to the training set for the subsequent iteration, whereas the rest was incorporated into the validation set (see Figure 1c). This process was repeated for four iterations. To ensure convergence, we extracted additional 200 snapshots using the final NequIP model to form a test set, against which we validated that the force and energy RMSEs leveled off below a desired threshold (see Figure 1d).

**Figure 2:**
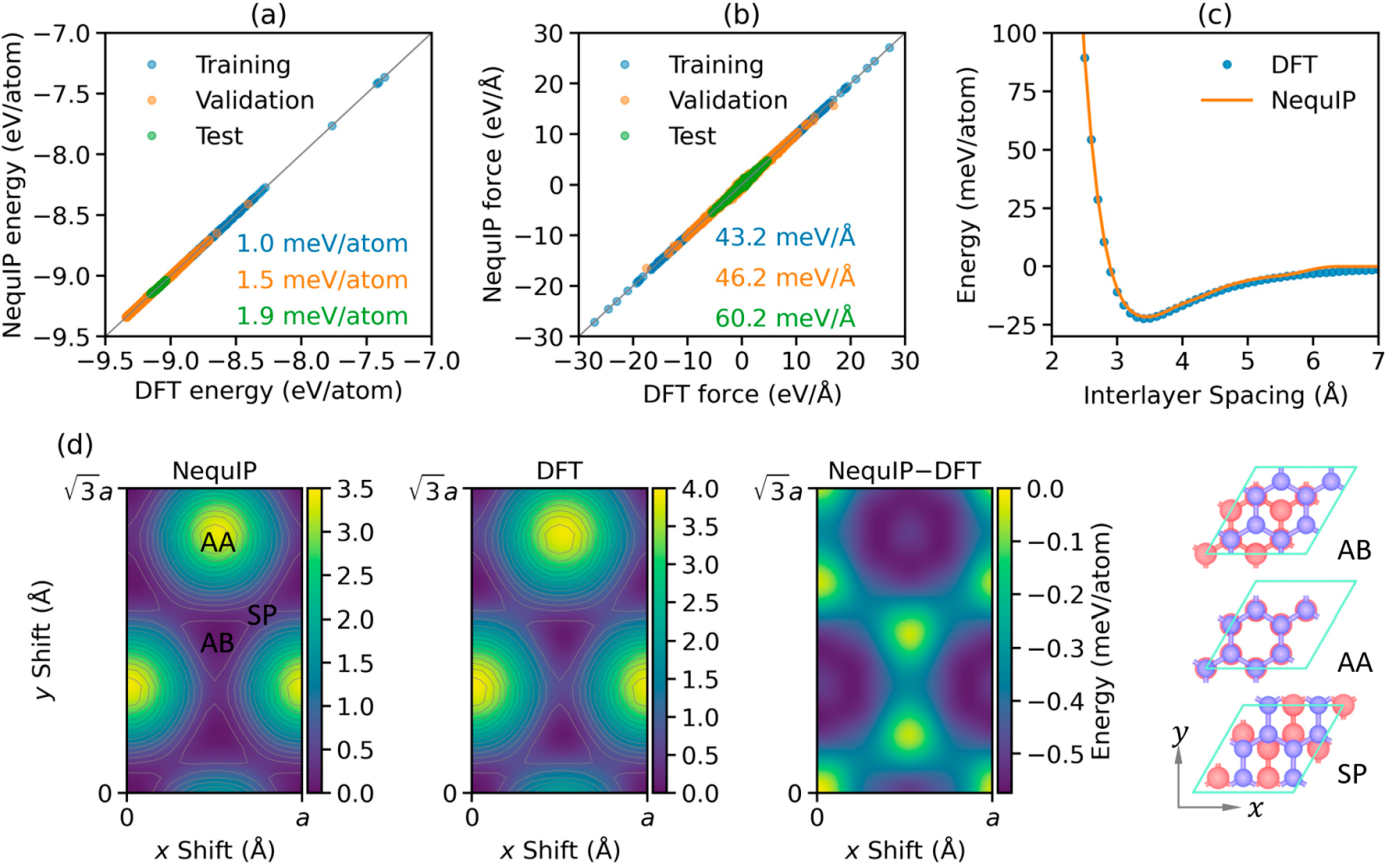
Comparisons between NequIP predictions and DFT calculations. Parity plots for (a) total energies and (b) atomic forces obtained for the training (blue), validation (orange), and test (green) data sets. (c) DFT (blue) and NequIP (orange) binding energy curves for AB stacked bilayer graphene. (d) NequIP (left) and DFT (middle) sliding PES for bilayer graphene, and their difference (right). The DFT and NequIP energy origins are set to the total energy of the AB stacked bilayer, correspondingly. A fixed lateral lattice parameter of *a* = 2.46 Å is used, and the vertical atomic positions are allowed to relax at each interlayer stacking. The AB, AA, and SP stacking modes, whose positions are marked on the NequIP PES map, are presented on the right.

As shown in Figure 2a,b, the final NequIP model achieves very high accuracy, with energy and force RMSE values lower than 2.0 meV/atom and 60.5 meV/Å, respectively, across all data sets. Furthermore, it captures well the PBE+MBD binding energy curve of AB stacked bilayer graphene (see Figure 2c) without requiring an explicit treatment of long-range dispersion interactions,^16,42,43^ and the corresponding shallow sliding potential energy landscape down to a deviation smaller than 0.6 meV/atom (see Figure 2d).

**Figure 3:**
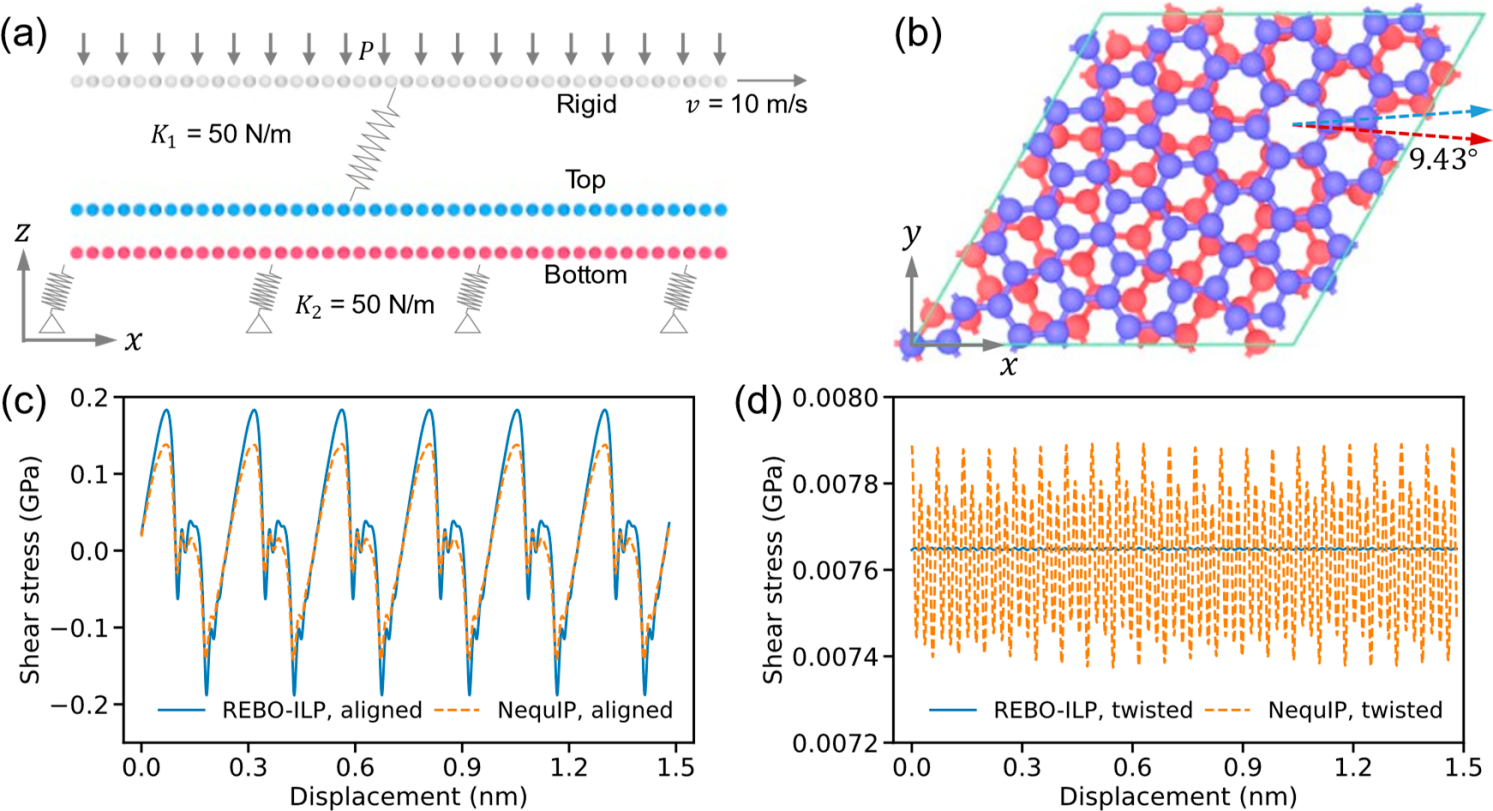
Friction simulations of the pristine (V_0_V_0_) bilayers. (a) Schematics of the simulation setup (see Methods for details). (b) Top view of the 9.43° twisted bilayer graphene considered in this work. REBO-ILP (blue) and NequIP (orange) shear-stress traces for the (c) aligned and (d) twisted graphene bilayers were obtained at a temperature of 0 K.

To test the reliability of the developed NequIP for describing sliding dynamics in layered interfaces, we validated it against well-established interlayer potential (ILP)-based MD simulations of aligned and twisted pristine bilayer graphene.^20^ The simulation setup presented in Figure 3a,b comprised two flexible graphene layers (red and blue spheres). The intra- and interlayer interactions were described either using the REBO^44^ and ILP,^45^ respectively, or using the developed NequIP. The atoms of the bottom layer were anchored by harmonic springs of stiffness 50 N/m to their original positions, mimicking a substrate of about two layers thickness (see SI section S4 for details). The top layer atoms were laterally driven along the zigzag direction by a rigid stage (a duplicate of the initial top layer structure) at a lateral velocity of 10 m/s via springs of the same stiffness. Periodic boundary conditions were used in the lateral directions, whereas free boundary conditions were applied in the out-of-plane direction. To model the effect of a normal load, a vertical force was applied to every atom in the rigid layer, thereby creating a normal pressure *P* transmitted to the top graphene layer through the harmonic springs. More details are given in the Methods section.

As shown in Figure 3c, the Rebo-ILP and NequIP shear-stress traces of the aligned interface show qualitatively the same stick–slip patterns with a maximal relative difference of 24.5% (0.05 GPa) at the peak positions. This difference can be partially attributed to the fact that the ILP was fitted against HSE (Heyd-Scuseria-Ernzerhof^46^ density functional approximation) + MBD reference DFT data, whereas the NequIP was trained at the PBE+MBD level of theory (see SI section S5). Conversely, for the 9.43° twisted bilayer, both Rebo-ILP and NequIP predict extremely low shear-stresses with the same average value (see Figure 3d). While the latter predicts larger oscillations, the overall difference between the two approaches is of the order of 1.7% (0.12 MPa), which is meaningless considering the overall extremely low shear stresses involved.

**Figure 4:**
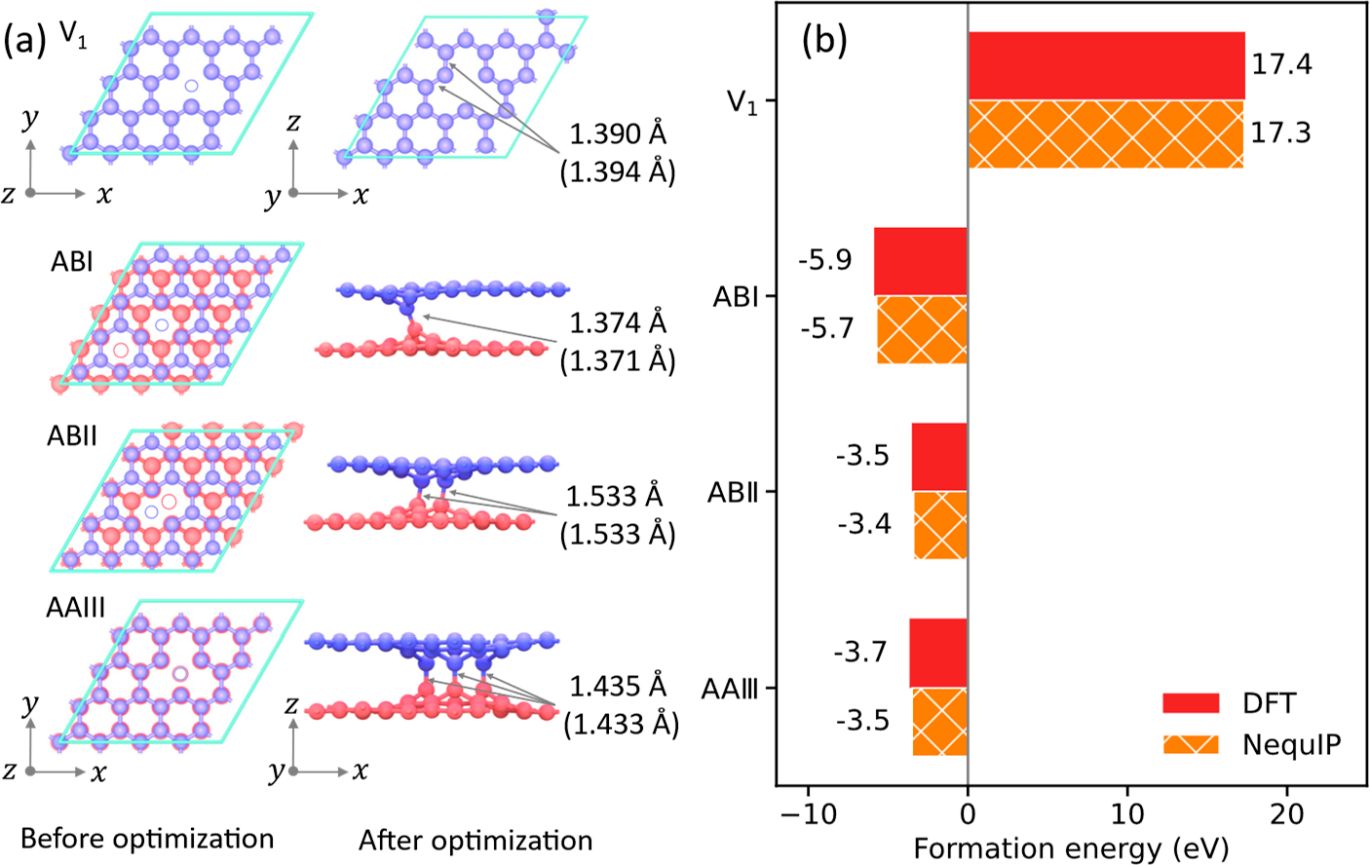
Comparisons of (a) the atomic structure and (b) the formation energy of defected graphene, as predicted by DFT and NequIP from independent optimizations, respectively. The bond lengths of the dangling atoms in the V_1_ layer and the interlayer bond lengths in bilayer defected graphene, as predicted by NequIP (and DFT), are compared in panel (a).

Having established the suitability of the developed NequIP machinery for studying the static and sliding dynamics of layered interfaces, we now turn to applying it to the case of defected graphene contacts. We start by evaluating energetic considerations of interlayer bond formation.^42,47−49^ To that end, we constructed three V_1_V_1_ model systems, differing by the relative lateral position of the two vacancies (see Figure 4a), as well as a monolayer defected graphene with one vacancy (V_1_), and relaxed them using either DFT or NequIP. Figure 4b compares the corresponding defect formation energy of the V_1_ monolayer and the bond formation energy of three V_1_V_1_ bilayers. The defect formation energy of the V_1_ monolayer was calculated as the energy difference between the relaxed V_1_ structure and pristine monolayer graphene, whereas the bond formation energy of the three V_1_V_1_ bilayers was defined as the energy difference between their relaxed structures and twice the energy of a relaxed V_1_ monolayer (see Methods for further computational details). The overall difference between the two computational approaches is typically smaller than 5.5%. Additionally, the structures optimized using NequIP closely align with those from DFT optimization, exhibiting atomic position deviations of less than 0.3 Å and even smaller interlayer bond deviations (<0.005 Å) as shown in Figure 4a.

Before moving on to applying NequIP to the case of reactive sliding dynamics, we have evaluated its performance against other reactive MLPs (including the deep potential (DP),^50^ neuroevolution potential (NEP),^36,51^ Gaussian approximation potential (GAP),^33^ and hNN-Gr_χ_^42^) and traditional potentials (including AIREBO,^52^ Tersoff,^53^ LCBOP,^54^ and ReaxFF^55^) for the case of pristine bilayer sliding and defected bilayer binding energetics. Our findings, presented in SI sections S6 and S7, indicate that while being the relatively computationally demanding of all approaches considered, the newly developed NequIP is also the most accurate to describe defected graphitic interfaces (see SI Figure S13).

**Figure 5:**
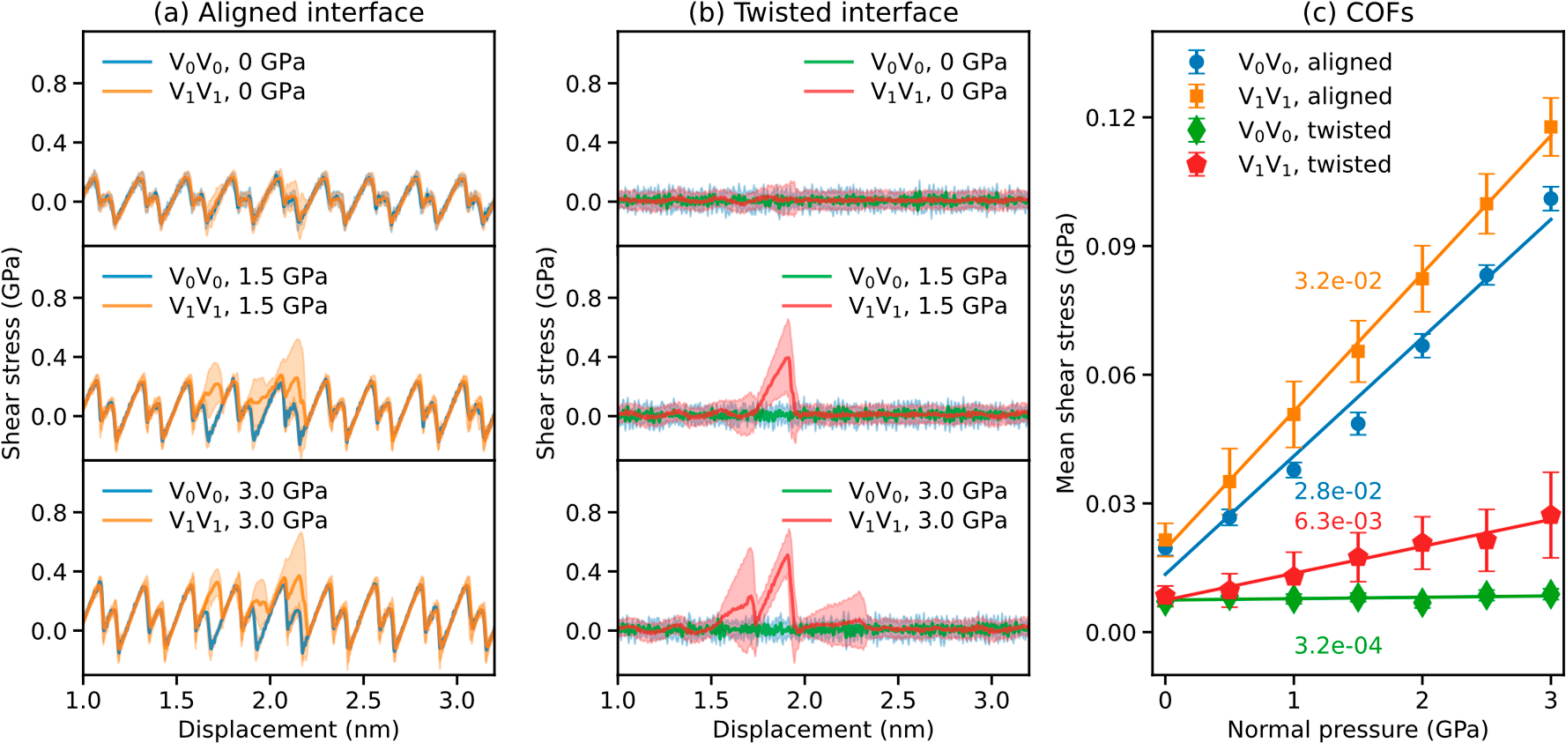
Room temperature sliding dynamics simulations of defected bilayer graphene. (a) Shear-stress traces of the aligned V_1_V_1_ bilayers (orange) under normal pressures of 0 (top panel), 1.5 (middle panel), and 3 (bottom panel) GPa. Results of the corresponding pristine interface (V_0_V_0_) are shown for comparison in blue. The shaded areas represent the standard deviation calculated for 100 trajectories. (b) Same as panel (a) for the 9.43° twisted interfaces. (c) Mean shear stress (averaged over a displacement of 5.2 nm of the ensemble-averaged trace) as a function of normal pressure for the aligned (orange) and twisted (red) V_1_V_1_ interfaces. The defect density for the aligned and twisted interfaces is 0.24 and 0.34%, respectively. Results for the corresponding pristine (V_0_V_0_) interfaces are presented in blue and green, respectively. The COFs extracted from the slopes of the linear fitting curves are marked near each line.

**Figure 6:**
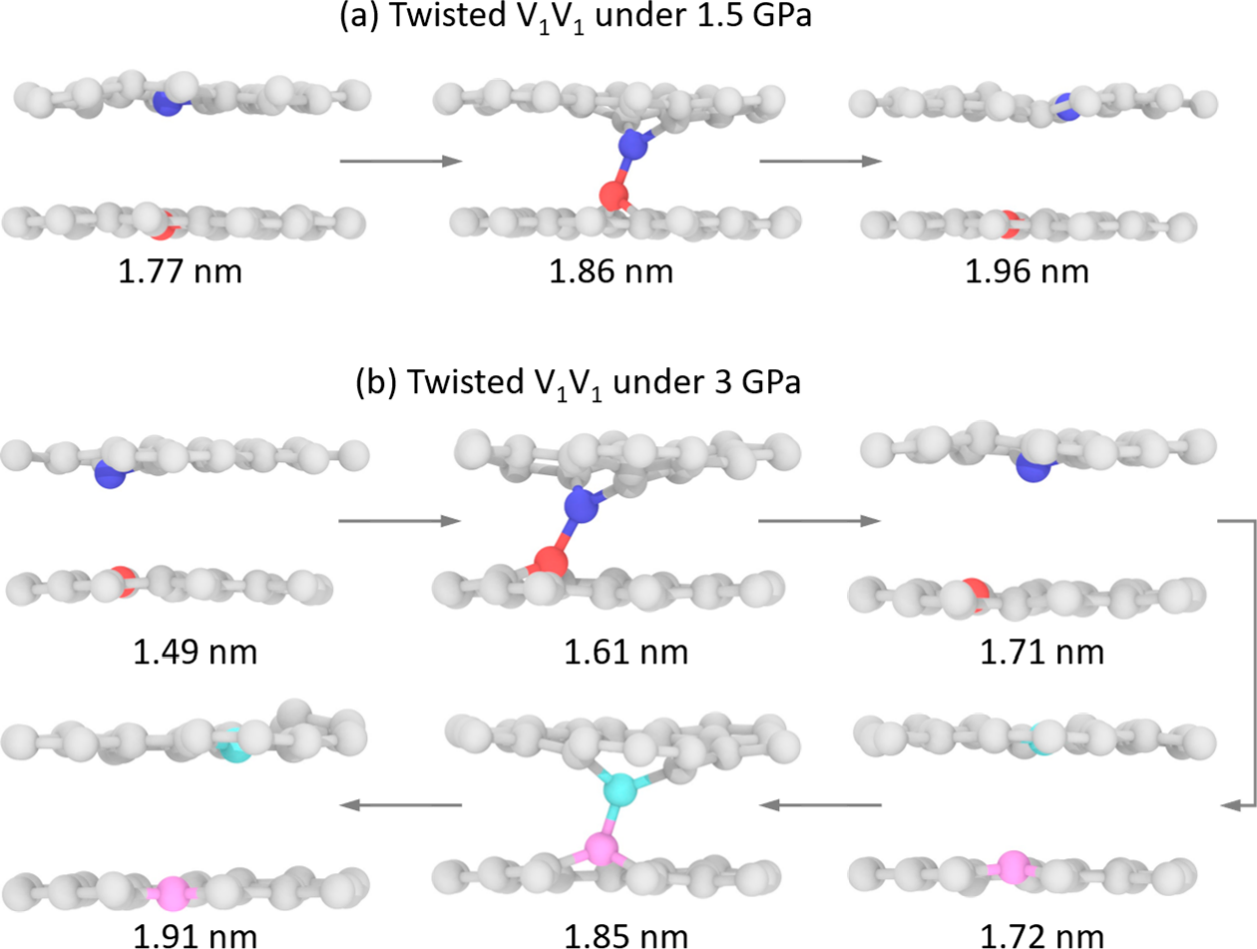
Demonstration of single and consecutive bond formation and rupture events. (a) Snapshots from a room temperature trajectory of a twisted V_1_V_1_ system, under an external pressure of 1.5 GPa, demonstrating a single bond formation and rupture event during sliding. (b) Same as panel (a) but under an external pressure of 3 GPa, where the system exhibits two consecutive events of bond formation and rupture. Only atoms in the vicinity of the defects are presented, and atoms involved in interlayer bonding are highlighted by colors. Lateral displacements are annotated below each snapshot.

Figure 5a illustrates three representative shear-stress traces of the aligned V_1_V_1_ bilayer (orange) under different normal loads. In the absence of an external load, the ensemble-averaged force trace of the defected interface resembles that of the pristine one (blue), indicating minor interlayer bonding effects on the sliding dynamics. Indeed, by examining the individual trajectories, we found that only 12 out of 100 of the 520 ps trajectories exhibited interlayer bonding. Here, interlayer bond formation was defined as a state where the distance between two atoms on adjacent layers is less than 1.8 Å. A similar behavior was found also for the twisted interface under zero normal load (see Figure 5b), with a bond formation probability of 3%. At a moderate pressure of 1.5 GPa (middle panel of Figure 5a), the interlayer binding probability increases (94% of all trajectories exhibit binding), resulting in substantial deviations of the V_1_V_1_ trajectory from the corresponding V_0_V_0_ one at a displacement of ∼1.85 nm, where the two defects are eclipsed. Notably, for the twisted interface under the same normal load, the friction trace (which in the absence of defects is superlubric) is found to be completely dominated by interlayer binding events with a bond formation probability of 80% (middle panel of Figure 5b, see also Figure 6a). A qualitatively similar picture arises under a normal load of 3 GPa (see lower panels of Figure 5a,b), with an increase of bond formation probability to 100%, a wider displacement range along which interlayer binding can occur, and the observation of multiple events of bond formation and rupture (Figure 6b). The above results indicate a dual contribution to the friction of defected interfaces: a physical contribution resulting from the corrugation of the sliding PES of the pristine lattice and a chemical contribution due to interlayer bonding.

These effects are naturally manifested in the friction coefficients of the different interfaces. Figure 5c presents the averaged shear-stress dependence on the external normal load. First, as expected, the misaligned twisted interfaces present consistently lower shear-stress than their aligned counterparts. The inclusion of defects increases the friction coefficient of the interface due to interlayer binding effects, where the relative increase is found to be considerably more dramatic for the twisted interface, which in the absence of defects, exhibits superlubric characteristics (COF of 3.2 × 10^–4^). Nonetheless, under the defect density considered herein, interlayer binding does not completely eliminate the superlubric nature of the twisted interface (the COF increases to 6.3 × 10^–3^). We note that under ultrahigh external pressures (>10 GPa) intermittent interlayer covalent binding is expected to occur also in pristine graphene interfaces via, e.g., SP^3^ hybridization.^56−58^ Hence, one may expect elimination of superlubricity of twisted graphene interfaces at the ultra-high-pressure regime not only through increased Pauli repulsions but also due to temporary local covalent binding.

Overall, our simulations demonstrate that the friction coefficient of defected layered interfaces can be separated into two components, namely, a physical and a chemical contribution. To demonstrate this, we write the coefficient as the ratio between the friction force and the normal force, COF = *F*_*r*_/*N*. The friction force is evaluated as an average over the lateral force trace, which can be split into two contributions: (i) nonbonded configurations (*F*_*r*_^NB^) that are dominated by physical drag similar to that of the pristine interface; and (ii) covalently bonded configurations (*F*_*r*_^B^) that are dominated by chemical drag, at sufficiently high defect densities. Correspondingly, the COF can be written as the sum of these contributions: COF = (*F*_*r*_^NB^ + *F*_*r*_^B^)/*N* = COF^phys^ + COF^Chem^. While the physical contributions of aligned and misaligned interfaces of similar surface areas differ by orders of magnitude, the chemical contributions are similar in both cases.

## Conclusions

The results presented above demonstrate the power of the developed MLP in the study of reactive dynamics at sliding layered interfaces. By providing a unified reliable treatment of intra- and interlayer interactions, we are able to reveal atomic scale mechanisms of complex bond formation and rupture, their dependence on normal and shear stresses, and their effect on friction and wear in superlubric sliding interfaces. To achieve this, we utilized the state-of-the-art GNN-based NequIP framework trained against quantum-mechanical DFT calculations via iterative learning cycles. For the pristine interfaces considered, the developed machinery was found to be ∼1–3 orders of magnitude slower than that based on physically motivated classical force-fields but still considerably faster to apply than standard ab initio molecular dynamics (AIMD, based on Born–Oppenheimer approximation) simulations (∼3–4 orders of magnitude faster, see SI Figure S13). Furthermore, the scaling of the latter with the system size is considerably more dramatic. Given that, when simulating the complex reactive sliding of defected layered interfaces, where dedicated classical force-fields are currently lacking, the developed MLP provides a desirable compromise between accuracy and computational burden. Finally, it should be noted that the same approach can be implemented for other defected layered interfaces, being homogeneous or heterogeneous. Notably, it is also applicable to bulk material interfaces where wear effects, which are very hard to study on the atomic level, are known to have a central tribological role.

## Methods

### GNN Potential Training

In this study, we use the NequIP^38^ scheme (version 0.5.6 with a total of 136,760 parameters) to develop a GNN potential for bilayer defected graphene. NequIP uses message-passing architectures and equivariant feature representations for enhanced data efficiency and accuracy.^38,59^ The former enables the interatomic interactions to propagate along the graph at each layer of the network. More specifically, the site energy of one atom is determined by the atomic environment of the neighbor atoms in adjacent layers through a series of convolutions within a predetermined cutoff distance (see Figure 1b). In the present study, we use a cutoff radius of 7 Å and four interaction layers. This setup, corresponding to an effective interaction cutoff distance of 28 Å, can account for long-range interactions. For equivariant feature representations, NequIP uses a set of irreducible representations of the rotational group O(3), each characterized by a rotation order *l. l* = 0,1, . . . denote scalars, vectors, and higher-order tensors, respectively. Here, we used a maximum rotation order of 1 with 32 features, thus maintaining a balance between high-order accuracy and the corresponding slower computational speed.^38,59^ These choices of cutoff radius, number of interaction layers, and rotation orders were previously tested and found to be adequate,^38,59^ as further verified by our extensive tests presented herein. To train the GNN, we employ the Adam optimizer^60^ with a learning rate of 0.005 and a batch size of 10. The total loss function is defined as a weighted average of the energy and atomic force errors on the training set. The termination of training was manually determined by monitoring the convergence of the loss for the validation set to prevent overfitting. Convergence is typically reached after 4,000–5,000 epochs. In total, we gathered 3,988 structures (with 143,437 atoms) and 4,467 structures (with 225,606 atoms) for the training and validation sets, respectively. All structures are provided in the shared data sets (see Data Availability Statement).

### MD Simulations

All MD simulations were carried out using the LAMMPS code (version 29 Sep 2021)^61^ with a dedicated interface to NequIP.^62^ All simulations were run at a temperature of 300 K using a Langevin thermostat^63^ in the canonical (NVT) ensemble. The thermostat was applied to all interface atoms in all directions with a damping parameter of 1 ps. The validity of this choice is demonstrated in SI section S9. A fixed time step of 1 fs was used throughout the simulations. We investigated two types of bilayer systems: V_0_V_0_ (pristine bilayers) and V_1_V_1_ (a single vacancy in each layer), each having two different stacking configurations (aligned and twisted). For the aligned interface, we used the AB stacking mode, yielding a size of 5.2 × 2.1 nm and containing 840 (838) atoms for the V_0_V_0_ (V_1_V_1_) bilayer. For the twisted interface, we constructed a bilayer system of a twist angle of 9.43°, to establish a relatively small hexagonal supercell, containing 148 atoms (Figure 3b), following the method described in ref (64). This hexagonal supercell was then expanded into a 5.2 × 1.5 nm rectangular supercell containing a total of 592 atoms for the V_0_V_0_ bilayer. To create the V_1_V_1_ bilayer supercell, we manually removed one atom in each layer of the corresponding V_0_V_0_ system to create a vacancy pair laterally spaced by ∼1.7 nm. More details on the atomic structure of all four bilayer systems are given in SI section S8. For structural visualization purposes, the OVITO package^65^ was used.

For the V_0_V_0_ bilayers, we calculated 10 independent trajectories for each simulation setup to compute the average force traces. Given the stochastic nature of interlayer bonding, we increased the number of independent trajectories to 100 for the V_1_V_1_ bilayers.

### DFT Calculations for Reference Data Generation

The DFT calculations were carried out using the PBE exchange-correlation density functional approximation^66^ in conjunction with a many-body dispersion (MBD) correction,^67^ as implemented in the Vienna ab initio simulation package (VASP).^68,69^ Single-point calculations were converged with an energy cutoff of 850 eV, using the projector augmented wave function (PAW) appaorch^70^ and a threshold of 10^–8^ eV for the electronic self-consistent loop. The out-of-plane supercell dimension is set at 50 Å to avoid spurious interactions between adjacent images. We sampled the in-plane Brillouin zone using a dense Γ-centered grid with a k-point density of 0.15/Å. The tetrahedron smearing method (ISMEAR = −5) was used for the total energy calculation.

### Defect Formation Energy Calculations

In this work, the formation energies of four defected structures were considered: (i) monolayer graphene with a single vacancy (denoted by V_1_); (ii) an AB-stacked V_1_V_1_ graphene bilayer, where the vacancies are laterally separated by 2.84 Å (denoted by ABI); (iii) an AB-stacked V_1_V_1_ bilayer, where the vacancies are laterally separated by 1.42 Å (denoted by ABII); and (iv) an AA-stacked V_1_V_1_ bilayer with eclipsed vacancies (denoted by AAIII). Here, I, II, and III denote the number of interlayer covalent bonds that these systems form following structural optimizations (Figure 4a). In these calculations, geometry optimization was performed for each structure separately using DFT and NequIP. In order to evaluate the formation energies of the interlayer bonded structures, we performed DFT geometry optimization of V_1_V_1_ bilayers, initially positioned at a subequilibrium interlayer distance of 2.0 Å. The optimization was performed using the VASP code, with the RMM-DIIS algorithm^71^ (IBRION = 1 keyword). Both supercell and atomic position optimizations were performed with an atomic force convergence criterion of 0.01 eV/Å. The optimized structures were then further relaxed using NequIP with the FIRE algorithm,^72^ as implemented in the atomic simulation environment (ASE) package.^73^

## Data Availability

The reference data sets and input files for NequIP, DP, and NEP are freely available at Zenodo (10.5281/zenodo.10374205). Additional data that support the findings of this study are available from the corresponding author upon reasonable request.

## Supporting Information Available

Reference structures in the initial data set; division of the initial data set using farthest point sampling; molecular dynamics simulations for iterative learning; effective spring stiffness for multilayer graphene stack simulations; energy barrier across different stacking modes; NEP and DP training; comparison with other traditional and machine-learning potentials; reactive sliding dynamics production simulations; validation of thermostatting approach (PDF)
