# Supplementary material for: Effect of Interlayer Bonding on Superlubric Sliding of Graphene Contacts: A Machine-Learning Potential Study
Source: ACS Nano. 2024 Mar 28;18(14):10133–41. doi: 10.1021/acsnano.3c13099 (PMC11008353; doi:10.1021/acsnano.3c13099)
Supplement: Supplementary file 1 — nn3c13099_si_001.pdf [file nn3c13099_si_001.pdf]

# Supporting Information

## Effect of Interlayer Bonding on Superlubric Sliding of Graphene Contacts: A Machine Learning Potential Study

Penghua Ying[1], Amir Natan[2], Oded Hod[1], Michael Urbakh[1]

1. Department of Physical Chemistry, School of Chemistry, The Raymond and Beverly Sackler Faculty of Exact Sciences and The Sackler Center for Computational Molecular and Materials Science, Tel Aviv University, Tel Aviv 6997801, Israel
2. Department of Physical Electronics, Tel-Aviv University, Tel-Aviv, 6997801, Israel

This supporting information document includes the following sections:

1. Reference structures in the initial dataset
2. Division of the initial dataset using farthest point sampling
3. Molecular dynamics simulations for iterative learning
4. Effective spring stiffness for multi-layer graphene stack simulations
5. Energy barrier across different stacking modes
6. NEP and DP training
7. Comparison with other traditional and machine learning potentials
8. Reactive sliding dynamics production simulations
9. Validation of thermostatting approach

## 1. Reference structures in the initial dataset

The initial dataset consisted of three bilayer systems, namely the $V_0V_0$ (pristine bilayers), $V_0V_1$ (a single vacancy on the top layer and a pristine bottom layer), and $V_1V_1$ (a single vacancy in each layer) with a total of 7,258 structures (369,043 atoms). The generation of configurations employed two methods: manual construction and molecular dynamics (MD) simulations, as described in the main text.

For the pristine $\mathbf{V_0V_0}$ bilayer system, the initial configurations consisted of four groups:

1. Binding configurations - for three distinct stacking modes - AA, AB, and SP, we use a primitive hexagonal cell consisting of four atoms (see Figure S1a). Within each stacking mode, rigidly vertically shifted structures were constructed with interlayer distances ranging from 2.0 Å to 10.0 Å in increments of 0.1 Å, yielding a total of 243 structures.
2. Sliding configurations - starting from an AB-stacked bilayer in a primitive hexagonal cell containing four atoms, we constructed a set of laterally shifted configurations by either rigid shifts of the top layer or flexible shifts, where the out of plane degree of freedom of each atom was allowed to relax. For rigid sliding, three different interlayer spacings (3.2 Å, 3.4 Å, and 3.6 Å) were employed, whereas for flexible sliding, an initial interlayer spacing of 3.4 Å was used. In each case, a grid of $21 \times 21$ shifted configurations (in the zigzag and armchair directions) was considered, yielding a total of 441 structures. This procedure led to a grand total of 1,764 structures.
3. Deformed configurations - for each of the binding configurations described in item #1 above, we constructed four deformed structures by applying random variations of up to 3% in the lattice vectors and of up to 0.1 Å in the atomic positions, thus generating a total of 972 deformed structures.
4. Molecular dynamics configurations - for the pristine $V_0V_0$ bilayer, we used the REBO[1]-ILP[2] force-fields to perform MD simulations (without sliding) at five different temperatures of 300, 600, 900, 1200, and 1500 K — employing the Nose-Hoover[3] thermostat. Here, a bilayer of 4×4 supercells, containing 64 atoms in total, was selected (see Figure S1b). Each simulation was run for 100 ps with a timestep of 1 fs. From these simulations, we sampled a total of 502 structures from the MD trajectories (202 structures from the 600 K and 1200 K simulations – 101 structures each, and 100 structures from each of the remaining various temperature simulations).

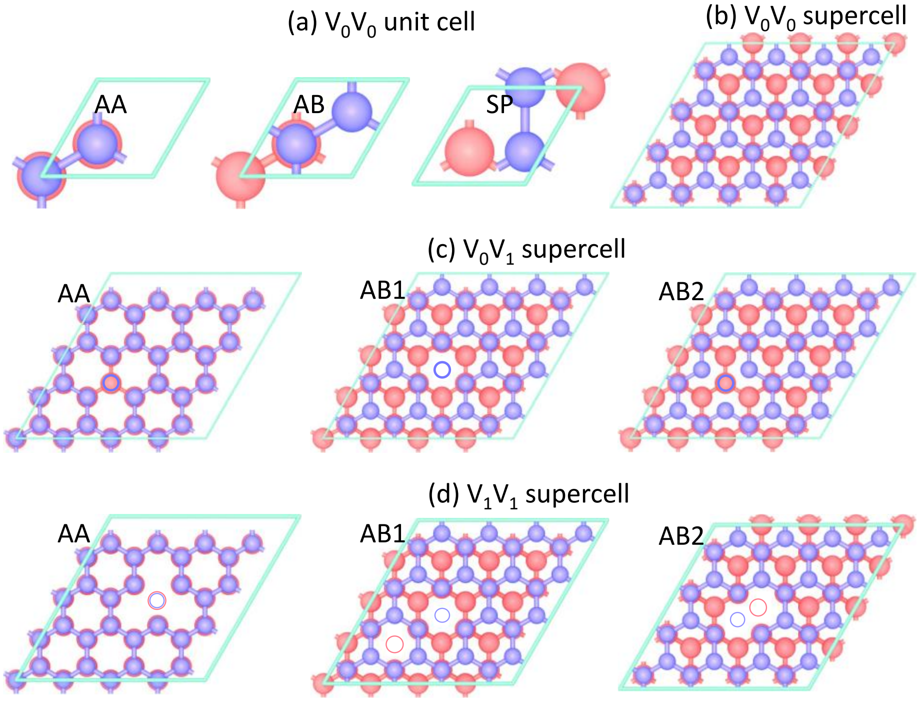


Figure S1. Atomistic structures of (a) a primitive 4 atom $V_0V_0$ unit cell; (b) a 64 atom $V_0V_0$ supercell; (c) a 63 atom $V_0V_1$ supercell; and (d) a 62 atom $V_1V_1$ supercell. The top layer atoms are depicted in blue, and the bottom layer atoms are depicted in red. Vacancies are represented by hollow circles.

For the $\mathbf{V_0V_1}$ bilayer system, the initial configurations consisted of two groups:

1. Relaxed configurations – three different stacking modes, each containing 63 atoms, have been considered: AA, AB1 (where the vacancy resides atop the center of a carbon hexagon in the bottom layer), and AB2 (where the vacancy is eclipsed with a carbon atom at the bottom layer) configurations (see Figure S1c). Structural optimization has been performed separately for each stacking configuration. To that end, we adopted the PBE exchange-correlation density functional approximation[4] with the Grimme D3 dispersion correction method (PBE+D3), [5] which is computationally less demanding than the PBE+MBD approach. An energy convergence criterion of $10^{-6}$ eV for the electronic self-consistent field (SCF) loop with a cutoff energy of 650 eV, and a force tolerance of 0.01 eV/Å for the ionic relaxation loop, were used. The in-plane Brillouin zone was sampled using a $\Gamma$-centered grid with a k-point density of 0.25/Å. Starting from the initial configuration, we selected one structure out of every five optimization steps.
2. Molecular dynamics configurations – for the standard ab-initio molecular dynamics (AIMD) simulations, we also used the PBE+D3 approach with an electronic SCF energy threshold of $10^{-5}$ eV, and an energy cutoff of 500 eV. The simulations were performed

with VASP[6, 7] (IBRION=0) using the isothermal ensemble at a temperature of 300 K, and a timestep of 1 fs. Here, the Brillouin zone was sampled only at the $\Gamma$ point. The three optimized structures of item #1 above were used as initial configurations for the MD simulations. Each simulation trajectory lasted 1000 propagation steps, out of which 20 structures have been evenly extracted.

With this, a total of 139 $V_0V_1$ structures have been obtained.

Similar to the $V_0V_1$ bilayer case the initial configurations of the **$V_1V_1$** bilayer consisted of relaxed and MD configurations each containing 62 atoms (see Figure S1d). To capture the dynamics of bond formation and breaking we considered several interlayer spacings and temperatures. The optimization was performed using the same approach as in the $V_0V_1$ case, where we considered three initial interlayer spacings (2.0 Å, 2.5 Å, and 3.0 Å) at each stacking mode and extracted the structure at each optimization step. The MD simulations also followed the $V_0V_1$ case now starting from the nine optimized structures and performed at temperatures of 300, 900, and 1500 K. From each of the 27 trajectories that were 1000 propagation steps long, we evenly extracted 100 structures.

With this, a total of 3638 $V_1V_1$ structures have been obtained.

## 2. Division of the initial dataset using farthest point sampling

In this study, we employed the farthest point sampling[8, 9] (FPS) method to split the initial dataset into a training subset and a validation subset. To that end, we first used the machinery of the neuroevolution potential (NEP) [10, 11] to convert the input configurations into descriptor vectors. Using these vectors, we performed a principal component analysis (PCA) [12, 13] that allowed us to extract a representative group of training configurations out of the entire initial dataset (see Figure S2), thus reducing the computational burden involved in the training stage of the NequIP potential. Using this approach, a total of 3299 structures (92375 atoms) were selected for the training set out of the initial 7258 structures (278357 atoms), using a minimal distance of 0.05 on the two-dimensional PCA plane.

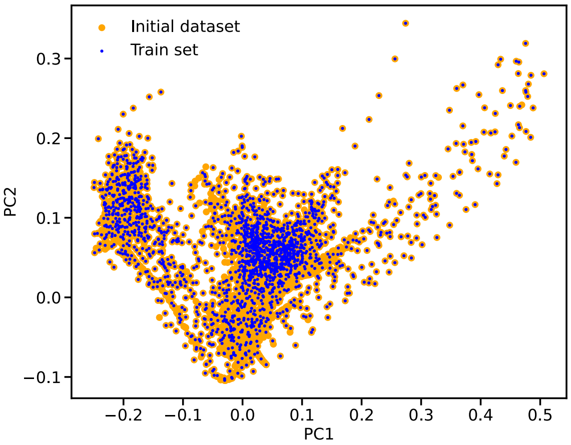


Figure S2. Two-dimensional PCA analysis used to extract the training set (blue points) out of the initial dataset (orange points).

## 3. Molecular dynamics simulations for iterative learning

As mentioned in the main text, during each cycle of the iterative learning algorithm applied to the $V_1V_1$ bilayer we performed sliding dynamics simulation to obtain additional training configurations. The simulation setup, described in Figure 3a and the Methods section of the main text, was adopted but with smaller model systems since the extracted structures were intended for further density functional theory (DFT) calculations. As depicted in Figure S3, the initial structure used in these MD simulations was an AB stacked $V_1V_1$ bilayer comprising of 78 atoms, where a vacancy pair was manually created by removing two atoms (one from each layer), laterally positioned 0.49 nm apart, corresponding to double the lattice parameter. This setup allows us to observe the formation of interlayer bonding as the two vacancies move close to each other under normal pressure loading. During each iteration, we carried out MD simulations at a temperature of 300 K for 123 ps (using a time step of 1 fs), with a sliding speed of 10 m/s in the zigzag direction.

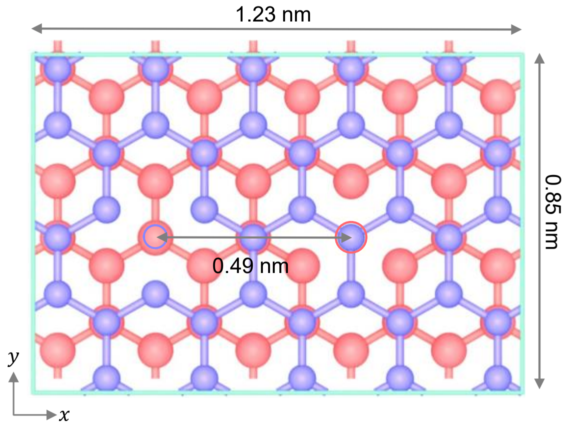


Figure S3. $V_1V_1$ bilayer model system used as an initial configuration for the MD simulations performed as part of the iterative learning process. Carbon atoms in the top layer are depicted in blue, those in the bottom layer are depicted in red, and vacancies are represented by hollow circles.

## 4. Effective spring stiffness for multi-layer graphene stack simulations

In Figure 3a of the main text, we used harmonic springs of stiffness of 50 N/m to anchor the substrate (bottom layer) atoms to their original positions. This stiffness was selected to mimic a bilayer substrate. To demonstrate this, we conducted a quasi-static sliding simulation along the zigzag direction of an aligned $V_0V_0$ bilayer model comprising of 840 atoms, starting from an initial AB-stacked configuration. During the simulation, the bottom layer atoms were kept fixed and only the vertical coordinate of the top layer atoms was allowed to relax following each displacement step. Figure S4 presents the lateral force acting on the center of mass of the top layer as a function of lateral displacement. A fit to the linear section of the force trace yields a stiffness (slope) of $k = 56.8\ \mathrm{N/m}$. For an $M$-layered graphene stack, there are $M - 1$ such interfaces that can be mimicked by $M - 1$ serial harmonic lateral springs (each of stiffness $k$). The effective stiffness of the stack can be estimated via $K_M = k/(M - 1)$. Using $K_M = 50\ \mathrm{N/m}$ we obtain M $\approx$ 2.

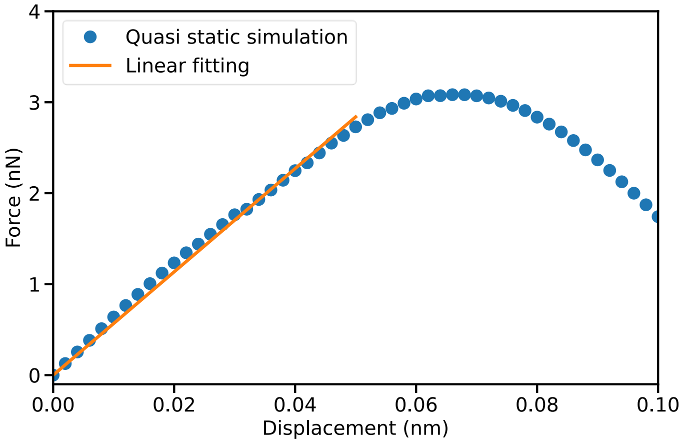


Figure S4. Lateral force (blue circles) acting on the center of mass of the top layer of a $V_0V_0$ bilayer, as a function of lateral displacement during NequIP quasi-static sliding simulations. Linear fit is presented by the orange line.

## 5. Energy barrier across different stacking modes

In Figure 3c of the main text, we observe a slight difference in the ILP and NequIP force traces of the aligned pristine interface. As demonstrated in Figure. S5a-c, the rigid sliding potential energy surfaces (PESs) obtained by ILP, NequIP, and PBE-MBD are in qualitative agreement. However, ILP predicts a higher energy corrugation of 7 meV/atom compared to 3.5 meV/atom and 4.0 meV/atom predicted by NequIP and PBE-MBD, respectively. We attributed this discrepancy mainly to the different levels of DFT theory employed as reference datasets for the two potentials. Specifically, NequIP is based on PBE-MBD reference calculations, whereas ILP is based on reference data obtained at the HSE-MBD level of theory.

Figure S5d compares the ILP, NequIP, and PBE-MBD sliding energy profiles along the armchair direction with representative HSE-MBD results obtained at the high symmetry AA, AB, and SP stacking modes. Notably, the NequIP profile (full blue line) agrees well with the PBE-MBD results (blue circles, differences smaller than 0.5 meV/atom). The ILP profile (orange dashed line) matches the HSE-MBD reference data (orange squares, which is somewhat higher than that predicted by PBE+MBD) for the AB to SP barrier and overestimates the AB to AA barrier by an acceptable value of 2.1 meV/atom.

Both ILP and HSE-MBD predict a higher energy for the AA stacking mode than NequIP and PBE-MBD, indicating the consistency between the potentials and their corresponding DFT reference data. It's worth noting that both ILP and NequIP underestimate the energy of the SP stacking mode, as seen in the inset of Figure S5d.

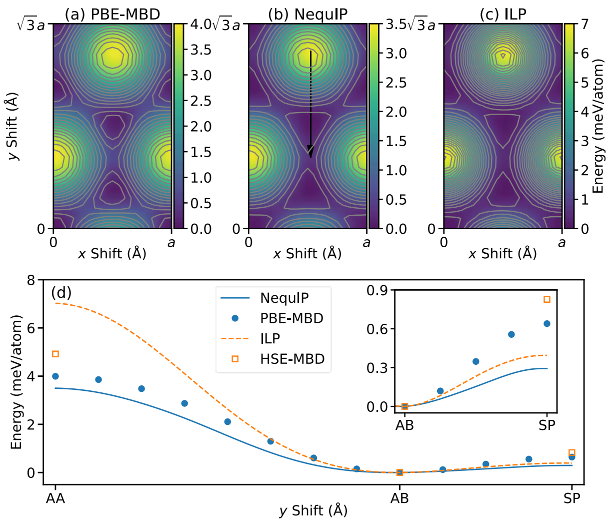


Figure S5. Sliding PESs of pristine bilayer graphene as predicted by (a) PBE-MBD, (b) NequIP, and (c) ILP. Panel (d) presents the energy profile along the direction of the black arrow marked in panel b, as predicted by the three methods, compared to HSE-MBD results for high symmetry stacking modes. The inset in (d) provides a zoomed-in view of the profile along the AB to SP path. All energy values are reported relative to the AB stacking mode of the corresponding calculation method.

## 6. NEP and DP training

For comparison purposes, apart from NequIP, we have also used the NequIP reference dataset to train the NEP and the deep potential (DP[14]) machine learning force-fields. The input and output training files are provided in the Zendo repository (10.5281/zenodo.10374205).

For NEP, we used the NEP3 framework[10, 11] implemented in the GPUMD package (version 3.7).[15] For both the radial and angular descriptor components, we used 13 radial functions, each being a linear combination of 13 Chebyshev polynomials. The neighbor list cutoff distance for the radius and angular descriptor components was set to 7 Å (which is same with NequIP) and 4 Å, respectively. For the angular components, we used both three-body and four-body correlations up to $l = 4$ and $l = 2$, respectively. The number of neurons in the hidden layer was chosen to be 80. For the hyperparameters related to the loss function, the weights of both the $\mathcal{L}_1$ and $\mathcal{L}_2$ norms were set to 0.05, and the weights of both energy and force terms were set to 1.0 (as in Ref 11). The neural network was trained over 500,000 generations using the natural evolution strategy[16] with a population size of 50. Figure S6a and b present the energy and forces correlation plots, respectively, obtained using the trained NEP.

For DP, we employed the DeePMD-kit package (version 2.2.2)[14] with a hybrid descriptor combining se_e2_a (with a cutoff of 6 Å) and se_e3 (with a cutoff of 4 Å) to describe two- and three-body interactions, respectively.[17] For the se_e2_a descriptors, the embedding net dimensions were (25, 50, 100), and for the se_e3 descriptors they were (20, 40, 80). The fitting net dimensions were set to (240, 240, 240) for both descriptors. The initial weighting parameters for the energy, forces, and virials were set to 0.02, 1000, and 0.01 respectively, which are linearly adjusted to 1, 1, and 0.1 during the training process. The training involved 7,594,000 steps with an exponentially reducing learning rate (from $10^{-3}$ to $10^{-8}$). Figure S6c and d present the energy and forces correlation plots, respectively, obtained using the trained DP.

In contrast to NequIP (energy and force root mean square errors (RMSEs) less than 1.5 meV/atom and 50 meV/Å for both training and validation sets, respectively, see Figure 2a of main text), both DP and NEP exhibit significantly higher errors in predicting energies and atomic forces for both training and validation sets, with energy RMSEs exceeding 3.5 meV/atom and force RMSEs surpassing 110 meV/Å, as demonstrated in Figure S6.

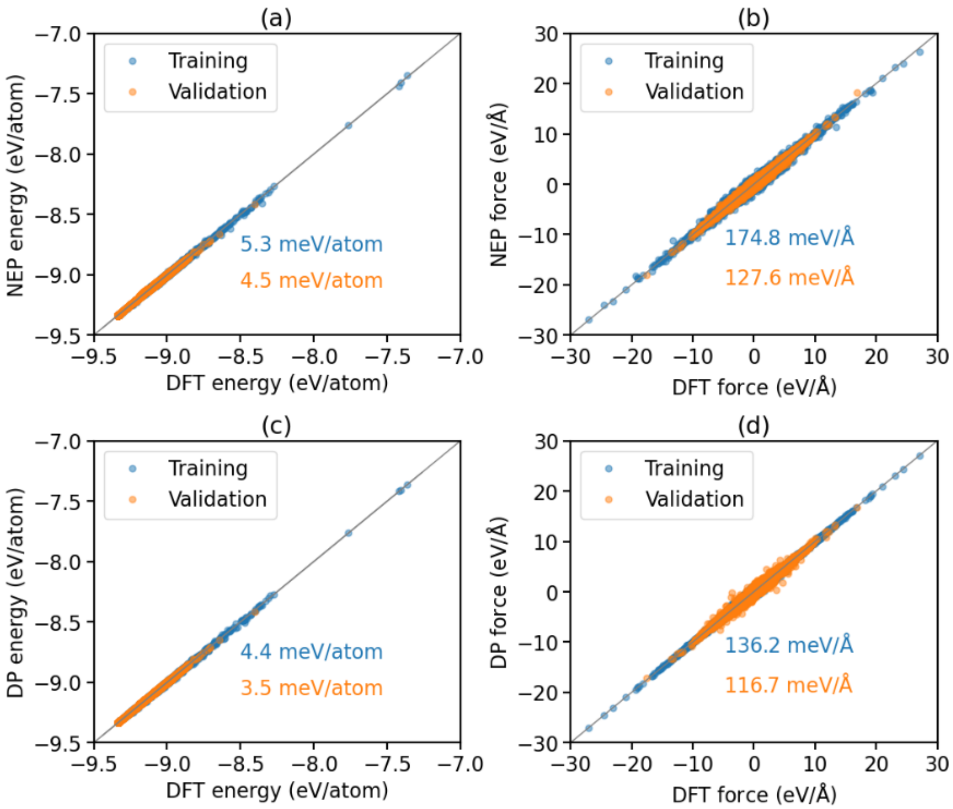


Figure S6. (a) energy and (b) atomic force NEP correlation maps (against DFT reference data) obtained for the training (blue) and validation (orange) sets. (c) and (d) are the same as (a) and (b) but for DP.

## 7. Comparison with other traditional and machine learning potentials

In the main text, we have focused on the reactive NequIP approach for defected interlayer graphene sliding. One may wonder whether less computationally demanding machine learning potentials (MLPs) or even existing reactive traditional potentials could be harnessed for this task, reducing the overall computational burden. To evaluate this, we have considered additional four MLPs and four traditional potentials. The MLPs include DP,[14] NEP,[10, 11] the Gaussian approximation potential (GAP-2020),[18, 19] and a hybrid neural network potential for graphene systems (referred to as hNN-$Gr_\chi$).[20] Out of these, DP and NEP were trained using the final training set of NequIP (see section S6 above for details), whereas for GAP-2020 and hNN-$Gr_\chi$, which were developed as general purpose potentials for carbon-based and graphitic systems, respectively, the original parameterizations were adopted. The considered reactive traditional potentials included AIREBO,[21] Tersoff,[22] LCBOP,[23] and ReaxFF,[24] all of which were designed to treat graphitic systems and adopted with their original parameterizations in the present study.

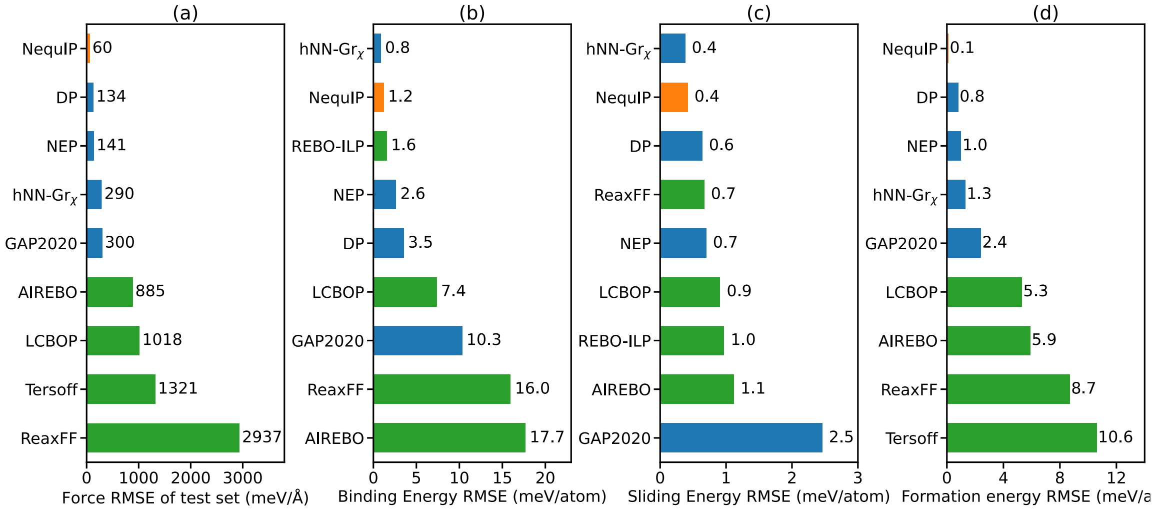


Figure S7. RMSE values of (a) forces, (b) binding energies, (c) sliding energies, and (d) formation energies, calculated for the NequIP (orange), four additional MLPs (blue), and four additional traditional potentials (green) against reference DFT calculations. Due to the absence of long-range van der Waals interactions, the Tersoff potential is excluded from the binding energy and sliding potential RMSE comparisons.

Figure S7 compares the RMSEs of the forces, and binding, sliding, and formation energies calculated for the various potentials against reference DFT calculations. For the forces (Figure S7a) we used the test set described in Figure 1c of the main text; for the binding (Figure S7b) and sliding (Figure S7c) energies we used the pristine bilayer configurations presented in Figure 2b, c of the main text; and for the formation energies (Figure S7d) we used the defected graphene structures presented in the Methods section (see also Figure

4a) of the main text. Generally, the MLPs perform better than the traditional potentials (but at a higher computational cost). Out of all MLPs considered, NequIP seems to perform best, with a significant advantage when describing the forces. Notably, hNN-Gr$_\chi$, which benefits from an analytical term designed for describing long-range dispersion, [20] performs well for pristine bilayer graphene binding and sliding energies. Nonetheless, it presents a considerable deviation in the forces for our test set that includes defected structures.

To rationalize the general outperformance of the MLPs over the traditional potentials, we present in Figure S8 force correlation maps plotted against DFT reference data for the test set. While all MLPs considered demonstrate correlation maps with clear diagonal character (top panels), the traditional force fields exhibit significant deviations from the diagonal. This indicates that the task of developing traditional potentials, capable of appropriately describing reactive sliding in defected layered interfaces, is extremely non-trivial.

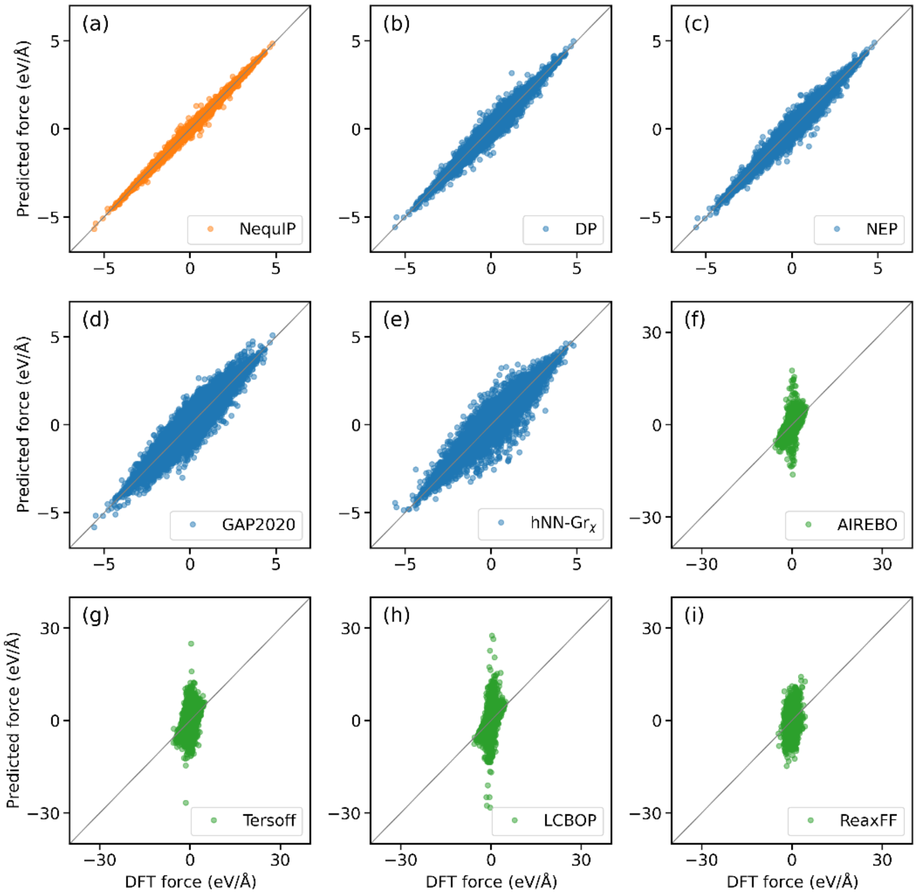


Figure S8. Force correlation maps for NequIP (orange), four additional MLPs (blue), and the four additional traditional potentials (green) considered, plotted against DFT reference data for the test set.

Focusing on binding, we plot in Figure S9 the binding energy curves obtained using the NequIP and additional eight potentials compared to the reference DFT results. By construction, all traditional force-fields give the correct physical binding profile, where AIREBO and ILP give excellent agreement with the reference data over the entire range, but LCBOP and ReaxFF exhibit significant deviations, especially in the vicinity of the equilibrium interlayer distance – the most physically relevant regime. In terms of the MLPs, both NEP and hNN-Gr$_\chi$ demonstrate accuracy similar to that of NequIP. Conversely, DP and GAP2020 yield quite irregular binding energy curves, where the latter presents an unphysical double minimum structure, consistent with previous observations. [25]

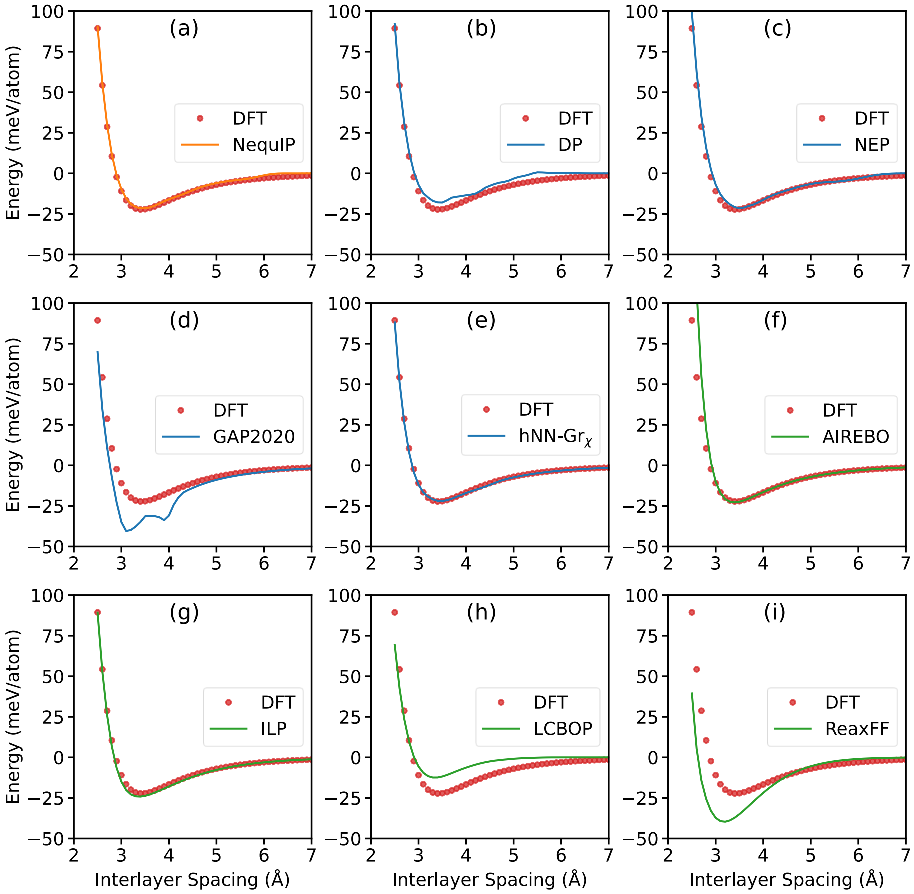


Figure S9: Binding energy curves of AB stacked pristine bilayer graphene calculated by rigid vertical shifts using the NequIP (orange), four additional MLPs (blue), and four additional traditional potentials (green) plotted in comparison to reference DFT calculations (red). The energy origin is set to the value of two infinitely separated graphene layers, obtained separately for each method.

Considering next the sliding physics, we plot in Figure S10 the sliding PESs, obtained using NequIP and the eight additional potentials. All traditional force-fields (AIREBO, ILP, LCBOP, and ReaxFF) yield the correct qualitative landscape. However, AIREBO and LCBOP significantly underestimate the sliding energy corrugation, whereas ILP and ReaxFF provide overestimate the reference DFT results. Here, one should recall that ILP was parameterized against HSE+MBD (rather than PBE+MBD) reference data (see Section S5 above). In terms of the additional MLPs (DP, NEP, GAP2020, and hNN-Gr$_\chi$), DP and GAP2020 are unable to reproduce the sliding energy landscape even qualitatively, whereas NEP and hNN-Gr$_\chi$ tend to overestimate and underestimate the sliding energy corrugation, respectively. Furthermore, NEP fails to describe the saddle point regions, and hNN-Gr$_\chi$ yields features that are too wide. Notably, NequIP provides the best agreement with the reference data out of all MLPs considered, including those trained herein.

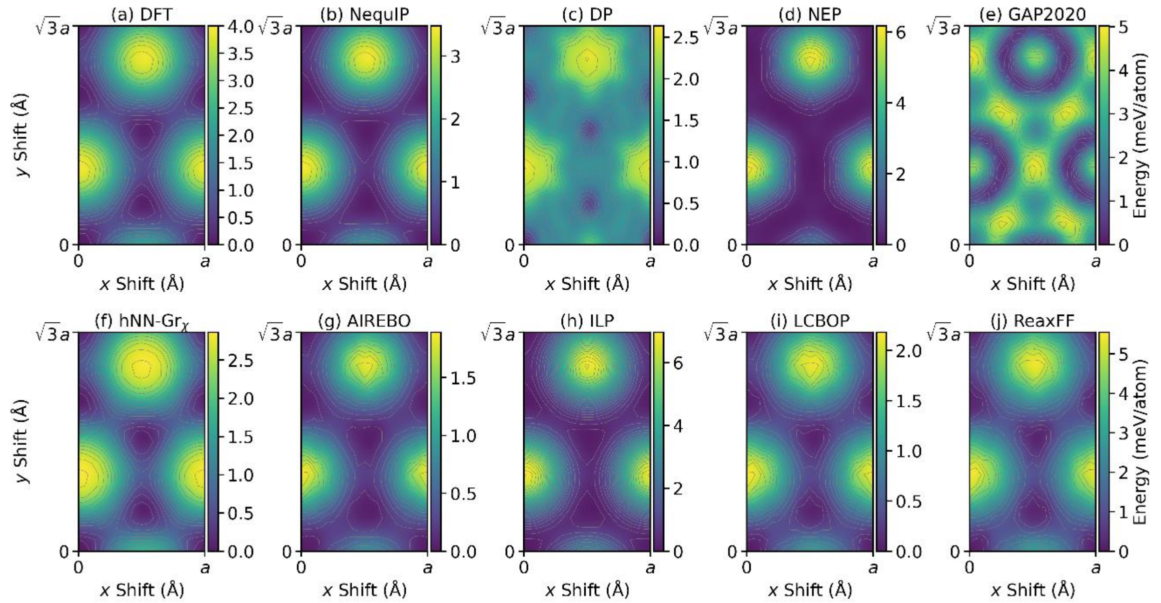


Figure S10. Sliding potential energy landscapes of bilayer graphene calculated using the (b) NequIP, (c-f) four additional MLPs, and (g-j) four additional traditional potentials plotted in comparison to (a) reference DFT calculations. The calculations are based on the DFT optimized configurations used to create Figure 2d of the main text. The energy origin is set to the value of AB stacked bilayer graphene, evaluated separately by each method for the same structure.

The last static property to consider is the formation energy of the defected structures. Figure S11 presents the formation energies of the defected structures considered in Figure 4a of the main text, as calculated using DFT (red) and NequIP (orange) with four additional MLPs (blue) and four additional traditional potentials (green). Altogether, the MLPs perform considerably better than the reactive traditional potentials, which show either significant quantitative and even qualitative (Tersoff) discrepancy with the reference data. Out of all MLPs considered, GAP2020 and hNN-Gr$_\chi$ provide the worst agreement with the

reference data, though generally better than the traditional potentials. NequIP, DP, and NEP perform similarly, where the former being the most accurate. This is not surprising, considering the fact that all three MLPs have been trained herein, where for the former we used an interactive learning scheme.

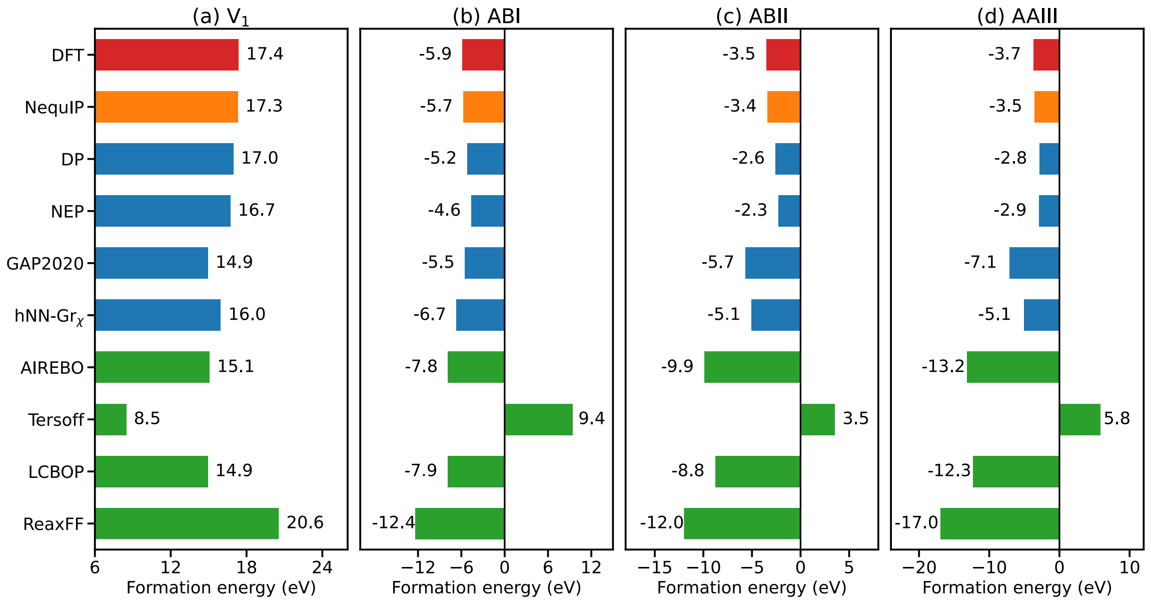

Figure S11: Comparison of the formation energies, predicted by DFT (red), NequIP (orange), and additional four MLPs (blue) and traditional potentials (green), for four defected structures: (a) $V_1$, (b) ABI, (c) ABII, and (d) AAIII (see Figure 4a of the main text).

To evaluate the dynamical performance of the various potentials, we present in Figure S12 the shear stress traces obtained from zero temperature (0K) dynamical simulations of pristine bilayer graphene sliding along the zigzag direction starting from the AB stacking mode. All simulations were performed using the LAMMPS package. [26] For the DP, NEP, GAP2020, and hNN-$Gr_\chi$ potentials, the DeePMD-kit, [27] NEP_CPU, [28] QUIP, [29] and KIM[30] packages were used as libraries called by the LAMMPS package, respectively. The simulation setup presented in Figure 3a of main text was adopted for all potentials.

From Figure S12 it becomes evident that both NequIP and hNN-$Gr_\chi$ provide good agreement with the reference REBO-ILP trace. NEP is able to capture the magnitude of the shear-stress variations and the overall periodicity but introduces shifts and high frequency variations that are absent in the reference trace. DP and GAP2020 demonstrate shear-stress traces that significantly deviate from the reference trace both quantitatively and qualitatively and cannot be used to describe the system dynamics in their present form. Notably, all traditional reactive potentials significantly underestimate the magnitude of the shear-stress variations obtained via REBO-ILP.

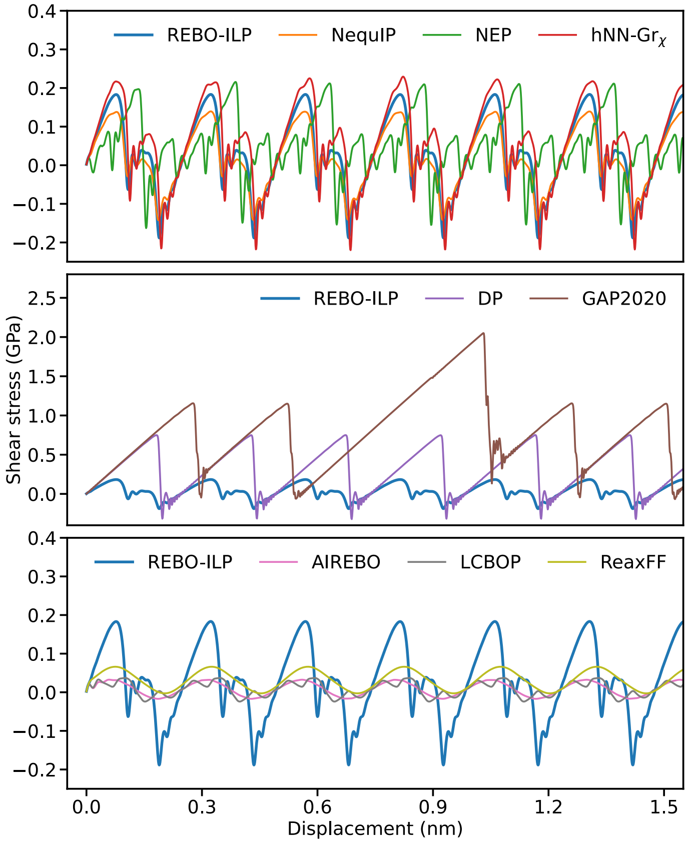


Figure S12. Dynamical shear stress traces of pristine bilayer graphene obtained using NequIP, NEP, and hNN-Gr$_\chi$ (top panel), DP and GAP2020 (middle panel), and the three additional traditional reactive potentials (bottom panel) considered, compared to the REBO-ILP trace.

Finally, in Figure S13, we compare the different potentials in terms of their accuracy and computational efficiency. As a measure of accuracy, we used the force RMSE evaluated by the potentials against reference DFT values obtained for the test set (see Figure S8). To evaluate the computational efficiency, we performed sliding dynamics simulations of pristine aligned bilayer graphene following the protocol described in Figure 3a of the main text. The simulations for all potentials started from the same initial AB stacked model system, consisting of 840 atoms (see Figure S14), and lasted for 520 ps. The elapsed wall time (divided by the number of atoms and number of time steps) was used as a measure of computational efficiency. For comparison we also performed a 100-steps standard AIMD simulation, on the same initial structure, using the PBE+D3 DFT density functional approximation as implemented in VASP. The simulation was performed with $\Gamma$-only sampling of the Brillouin zone, a planewave energy cutoff of 520 eV, and an energy threshold of $10^{-5}$ eV for the electronic self-consistent loop.

For NequIP and DP we used an Nvidia GeForce RTX 2080 Ti GPU card. For the other potentials and AIMD simulation, the 48 Intel(R) Xeon(R) Platinum 9242 CPU cores were used. We note that this is not an exhaustive comparison. First, we use a mixture of CPU and GPU platforms, where GPUs are used whenever available for a given potential or when they outperform CPUs. Second, we do not explore factors such as system size and the effect of interlayer bonding. Nonetheless, the comparison does provide important insights regarding the performance of the various approaches.

Clearly, all traditional reactive force-fields considered present overall low computational cost but at a price of significantly reduced accuracy. The MLPs are generally characterized by higher computational cost and increased accuracy. Notably, the developed NequIP provides a good compromise between accuracy and computational efficiency, being more than an order of magnitude more accurate than AIREBO and more than three orders of magnitude more efficient than AIMD.

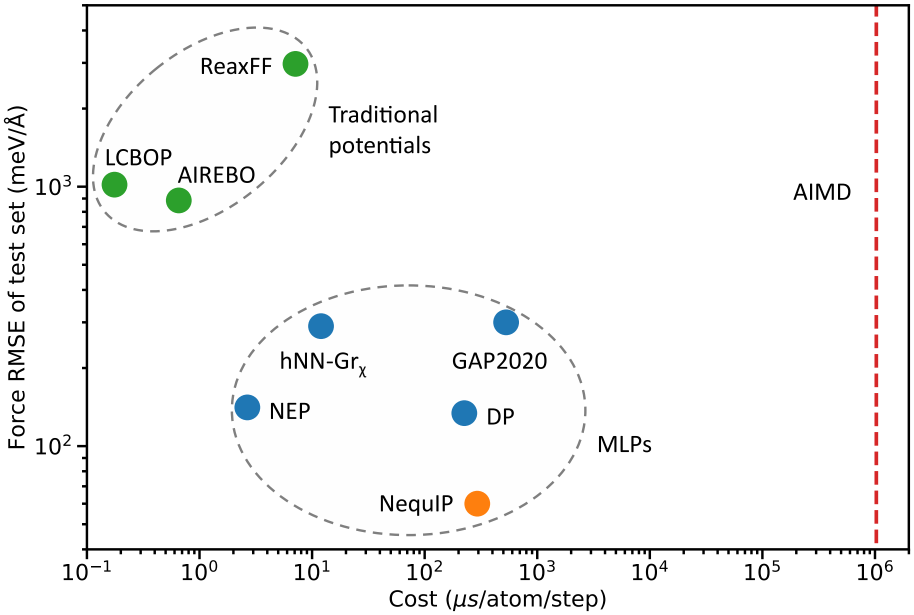


Figure S13. Ranking of the various potentials including NequIP (orange circle), additional four MLPs (blue circles), and additional four traditional potentials (green circles) considered in the present study, based on the test set force RMSE and dynamic simulation computational cost. Here, the computational speed of AIMD simulations (Red vertical line) is provided as a reference.

## 8. Reactive sliding dynamics production simulations

As a demonstration of the utility of the developed NequIP force-field we performed production MD simulations studying the effects of lattice defects and interlayer bonding on the frictional properties of graphitic interfaces. To that end, we considered aligned and twisted $V_1V_1$ bilayer systems and compared the results against those of the corresponding pristine $V_0V_0$ interfaces. Figure S14 shows the atomistic structures of aligned and twisted $V_1V_1$ bilayers, each mirroring their $V_0V_0$ counterparts, except for the intentional introduction of atomic vacancies. For the aligned $V_1V_1$ bilayer (top panel), we created two vacancies at a lateral distance of 1.72 nm (separated by seven lattice vectors). For the twisted $V_1V_1$ bilayer (bottom panel), a similar distance of 1.73 nm was chosen to introduce the vacancies. We introduce the vacancies such that upon sliding in the zigzag direction of the bottom layer they eventually arrive at an eclipsed configuration, where bond formation is most likely to occur. Room temperature (300 K) MD simulations have been performed for 516 and 518 ps for the aligned and twisted bilayer models, respectively, at a sliding velocity of 10 m/s, yielding the results presented in Figure 5 of the main text.

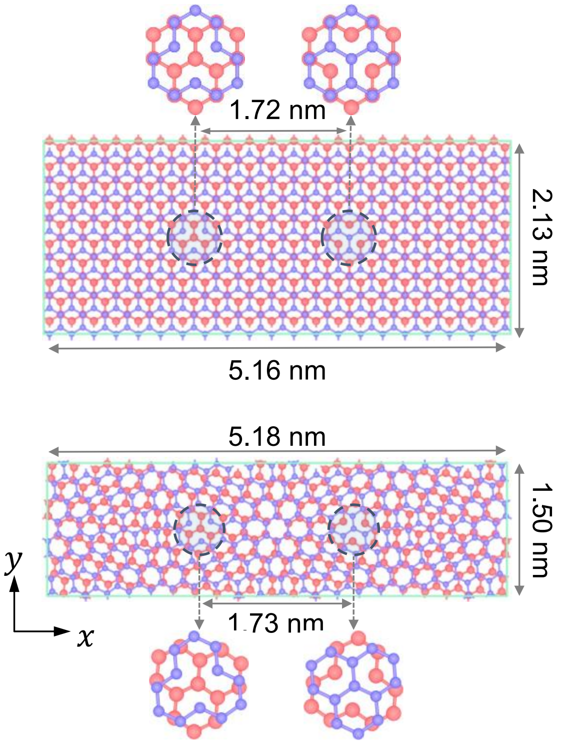


Figure S14. Top view of the aligned (top panel) and 9.43° twisted (bottom panel) $V_1V_1$ graphene bilayers used as initial configurations for the reactive sliding dynamics simulations. The insets highlight the vicinity of the vacancies.

## 9. Validation of thermostatting approach

The thermostatting approach adopted in the present study applies Langevin dynamics to all atoms at the sliding interface. To verify that the direct application of Langevin dynamics to the atoms participating in the covalent interlayer binding processes does not influence our conclusions, we have repeated some of our calculations while excluding the thermostat term from the dangling atoms in the defect regions (three in the top layer and three in the bottom layer, see the bottom panel of Figure S14). Ten additional independent random trajectories have been simulated for the twisted V1V1 bilayer under a normal pressure of 2.5 GPa at a temperature of 300 K.

Figure S15 compares the average shear stress trace obtained for the ten traces using the modified thermostatting approach (blue solid line) against that acquired for 100 traces using the original approach (orange dashed line). Notably, already for this small statistical ensemble of traces the modified approach is in excellent agreement with the original thermostatting procedure, leading us to the understanding that directly applying the Langevin thermostat to the sliding interface atoms, and specifically to those participating in the interlayer binding process, has minor effect on our conclusions.

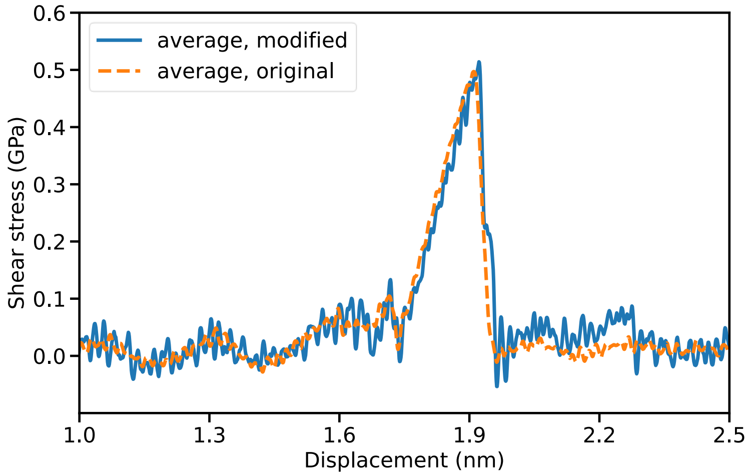


Figure S15. Comparison of the average shear stress traces obtained during the sliding dynamics simulations of the twisted $V_1V_1$ bilayer graphene at a temperature of 300 K and under a normal pressure of 2.5 GPa with the original (dashed orange line) and modified (solid blue line) thermostatting approach.

## References

(1) Brenner, D. W.; Shenderova, O. A.; Harrison, J. A.; Stuart, S. J.; Ni, B.; Sinnott, S. B. A second-generation reactive empirical bond order (REBO) potential energy expression for hydrocarbons. *Journal of Physics: Condensed Matter* **2002**, *14*, 783–802.
(2) Ouyang, W.; Mandelli, D.; Urbakh, M.; Hod, O. Nanoserpents: Graphene Nanoribbon Motion on Two-Dimensional Hexagonal Materials. *Nano Letters* **2018**, *18*, 6009-6016.
(3) Martyna, G. J.; Tobias, D. J.; Klein, M. L. Constant pressure molecular dynamics algorithms. *The Journal of Chemical Physics* **1994**, *101*, 4177-4189.
(4) Perdew, J. P.; Burke, K.; Ernzerhof, M. Generalized gradient approximation made simple. *Physical Review Letters* **1996**, *77*, 3865.
(5) Grimme, S.; Antony, J.; Ehrlich, S.; Krieg, H. A consistent and accurate ab initio parametrization of density functional dispersion correction (DFT-D) for the 94 elements H-Pu. *The Journal of Chemical Physics* **2010**, *132*, 154104.
(6) Kresse, G.; Furthmüller, J. Efficient iterative schemes for ab initio total-energy calculations using a plane-wave basis set. *Physical Review B* **1996**, *54*, 11169.
(7) Kresse, G.; Joubert, D. From ultrasoft pseudopotentials to the projector augmented-wave method. *Physical Review B* **1999**, *59*, 1758.
(8) Bartók, A. P.; De, S.; Poelking, C.; Bernstein, N.; Kermode, J. R.; Csányi, G.; Ceriotti, M. Machine learning unifies the modeling of materials and molecules. *Science Advances* **2017**, *3*, e1701816.
(9) De, S.; Bartok, A. P.; Csanyi, G.; Ceriotti, M. Comparing molecules and solids across structural and alchemical space. *Physical Chemistry Chemical Physics* **2016**, *18*, 13754-13769.
(10) Fan, Z.; Zeng, Z.; Zhang, C.; Wang, Y.; Song, K.; Dong, H.; Chen, Y.; Ala-Nissila, T. Neuroevolution machine learning potentials: Combining high accuracy and low cost in atomistic simulations and application to heat transport. *Physical Review B* **2021**, *104* (10). DOI: 10.1103/PhysRevB.104.104309.
(11) Fan, Z.; Wang, Y.; Ying, P.; Song, K.; Wang, J.; Wang, Y.; Zeng, Z.; Xu, K.; Lindgren, E.; Rahm, J. M.; et al. GPUMD: A package for constructing accurate machine-learned potentials and performing highly efficient atomistic simulations. *The Journal of Chemical Physics* **2022**, *157*, 114801.
(12) *https://scikit-learn.org/stable/modules/generated/sklearn.decomposition.PCA.html*. (accessed 2023-12-05).
(13) Pedregosa, F.; Varoquaux, G.; Gramfort, A.; Michel, V.; Thirion, B.; Grisel, O.; Blondel, M.; Prettenhofer, P.; Weiss, R.; Dubourg, V. Scikit-learn: Machine learning in Python. *Journal of Machine Learning Research* **2011**, *12*, 2825-2830.
(14) Wang, H.; Zhang, L.; Han, J.; Weinan, E. DeePMD-kit: A deep learning package for many-body potential energy representation and molecular dynamics. *Computer Physics Communications* **2018**, *228*, 178-184.
(15) Fan, Z.; Chen, W.; Vierimaa, V.; Harju, A. Efficient molecular dynamics simulations with many-body potentials on graphics processing units. *Computer Physics Communications* **2017**, *218*, 10-16. DOI: 10.1016/j.cpc.2017.05.003.
(16) Wierstra, D.; Schaul, T.; Glasmachers, T.; Sun, Y.; Peters, J.; Schmidhuber, J. Natural evolution strategies. *The Journal of Machine Learning Research* **2014**, *15*, 949-980.
(17) Zhang, L.; Han, J.; Wang, H.; Saidi, W.; Car, R. End-to-end symmetry preserving inter-atomic potential energy model for finite and extended systems. *Advances in neural information processing systems* **2018**, *31*.
(18) Bartók, A. P.; Payne, M. C.; Kondor, R.; Csányi, G. Gaussian approximation potentials: The accuracy of quantum mechanics, without the electrons. *Physical Review Letters* **2010**, *104*, 136403.
(19) Rowe, P.; Deringer, V. L.; Gasparotto, P.; Csanyi, G.; Michaelides, A. An accurate and transferable machine learning potential for carbon. *The Journal of Chemical Physics* **2020**, *153*, 034702.
(20) Wen, M.; Tadmor, E. B. Hybrid neural network potential for multilayer graphene. *Physical Review B* **2019**, *100*, 195419.
(21) Stuart, S. J.; Tutein, A. B.; Harrison, J. A. A reactive potential for hydrocarbons with intermolecular interactions. *The Journal of Chemical Physics* **2000**, *112*, 6472-6486.
(22) Kınacı, A.; Haskins, J. B.; Sevik, C.; Çağın, T. Thermal conductivity of BN-C nanostructures. *Physical Review B* **2012**, *86*, 115410.
(23) Los, J.; Fasolino, A. Intrinsic long-range bond-order potential for carbon: Performance in Monte Carlo simulations of graphitization. *Physical Review B* **2003**, *68*, 024107.
(24) Chenoweth, K.; Van Duin, A. C.; Goddard, W. A. ReaxFF reactive force field for molecular dynamics simulations of hydrocarbon oxidation. *The Journal of Physical Chemistry A* **2008**, *112*, 1040-1053.
(25) Qian, C.; McLean, B.; Hedman, D.; Ding, F. A comprehensive assessment of empirical potentials for carbon materials. *APL Materials* **2021**, *9*, 061102.
(26) Thompson, A. P.; Aktulga, H. M.; Berger, R.; Bolintineanu, D. S.; Brown, W. M.; Crozier, P. S.; in't Veld, P. J.; Kohlmeyer, A.; Moore, S. G.; Nguyen, T. D. LAMMPS-a flexible simulation tool for particle-based materials modeling at the atomic, meso, and continuum scales. *Computer Physics Communications* **2022**, *271*, 108171.
(27) *deepmd-kit.* https://github.com/deepmodeling/deepmd-kit. Accessed: 2023-08-07.

(28) *NEP_CPU*. https://github.com/brucefan1983/NEP_CPU. Accessed: 2023-08-07.
(29) *QUIP*. https://github.com/libAtoms/QUIP. Accessed: 2023-08-07.
(30) *kim-api*. https://github.com/openkim/kim-api. Accessed: 2023-08-07.
